# Reprogramming the immunological microenvironment through radiation and targeting Axl

**DOI:** 10.1038/ncomms13898

**Published:** 2016-12-23

**Authors:** Todd A. Aguilera, Marjan Rafat, Laura Castellini, Hussein Shehade, Mihalis S. Kariolis, Angela Bik-Yu Hui, Henning Stehr, Rie von Eyben, Dadi Jiang, Lesley G. Ellies, Albert C. Koong, Maximilian Diehn, Erinn B. Rankin, Edward E. Graves, Amato J. Giaccia

**Affiliations:** 1 Division of Radiation and Cancer Biology, Department of Radiation Oncology, 269 Campus Drive, Stanford University, Stanford, California 94305, USA; 2 Stanford Cancer Institute, 265 Campus Drive, Ste G2103, Stanford, California 94305, USA; 3 Department of Pathology, University of California San Diego, 9500 Gilman Drive, La Jolla, California 92093, USA

## Abstract

Increasing evidence suggests that ionizing radiation therapy (RT) in combination with checkpoint immunotherapy is highly effective in treating a subset of cancers. To better understand the limited responses to this combination we analysed the genetic, microenvironmental, and immune factors in tumours derived from a transgenic breast cancer model. We identified two tumours with similar growth characteristics but different RT responses primarily due to an antitumour immune response. The combination of RT and checkpoint immunotherapy resulted in cures in the responsive but not the unresponsive tumours. Profiling the tumours revealed that the Axl receptor tyrosine kinase is overexpressed in the unresponsive tumours, and Axl knockout resulted in slower growth and increased radiosensitivity. These changes were associated with a CD8^+^ T-cell response, which was improved in combination with checkpoint immunotherapy. These results suggest a novel role for Axl in suppressing antigen presentation through MHCI, and enhancing cytokine release, which promotes a suppressive myeloid microenvironment.

## Abstract (web summary)

Radiotherapy can enhance the antitumour immune response. Here, the authors show that resistance to radiation in breast cancer cells can be due to Axl expression that suppresses antigen presentation though MHCI, promotes NF-κB signalling, and enhances cytokine release promoting a suppressive myeloid microenvironment.

In recent years, cancer immunotherapy has demonstrated clinical benefit of targeting immune checkpoints that modulate immune-mediated tumour clearance. CTLA-4 and PD-1 are two negative regulatory immune checkpoints that modulate the T-cell response to antigens presented through the T-cell receptor and blocking antibodies (Ab) to immune checkpoints have led to multiple FDA (Food and Drug Administration) approvals since 2011 (ref. 1). Although single-agent immune checkpoint inhibitor therapy responses are limited to 10–30% of patients, responses can be dramatic in patients with metastatic disease, leading to extended survival^2^,^3^,^4^,^5^. Interest in combining radiation therapy (RT) with immune checkpoint therapy heightened after a case report by Postow *et al*.^6^ indicating that RT to one lesion triggered a systemic response in a melanoma patient who had previously progressed on the CTLA-4 Ab Ipilimumab. Similar observations have been published for a small number of patients, and both clinical and preclinical data have validated potential benefits for the combination of RT with CTLA-4 and PD-1 blockade^7^,^8^,^9^,^10^,^11^.

It is important to understand why many patients do not respond to checkpoint immunotherapy or its combination with RT. Factors that may impact efficacy of the combination include limited antigen presentation by tumour cells or antigen-presenting cells, or a tumour-promoting microenvironment that suppresses antitumour immune responses. Many preclinical models evaluating RT and immunotherapy have focused on immunogenic model antigens, thus bypassing these factors^12^,^13^. It is known that RT can enhance antigen presentation by increasing major histocompatibility complex class 1 (MHCI), but responses can be diminished through increased PD-L1 expression, thereby providing a rationale for combination therapy^9^,^14^,^15^. However, there is a need to identify factors that suppress the immune response, rendering RT or its combination with immune checkpoint therapy ineffective. For example, Stefani *et al*. recently identified the β-catenin signalling pathway that suppresses licensing of dendritic cells through chemokines in melanoma, and we propose that this pathway may also suppress responses to radiation and combination checkpoint therapy^16^. Alternatively, targeted therapies have been shown to have independent effects on the immunological compartment. For example, MET oncogene inhibition can promote tumour progression through inhibition of antitumour neutrophils, and PI3 kinase inhibitors can block myeloid suppressor cells, thus enhancing antitumour responses^17^,^18^. However, little is known about the cellular and microenvironmental factors that influence the immune stimulatory effects of RT.

In this study, we sought to identify tumour cell derived factors that suppress immunological responses and limit the RT response and its combination with checkpoint immunotherapy. Although some tumours are known to be more radio-responsive than others, comparisons have limited utility due to genetic diversity between different tumour types. Therefore, we evaluated immune responses after RT using clonal tumours derived from the same parental transgenic mouse model, which would allow comparisons between tumours in a syngeneic setting. We focused on two tumour clones with unique immune responses after RT. These clones represent differences in responses often found in tumours from human patients. When evaluating differences between the tumours, we identified Axl, a TAM (Tyro3/Axl/Mer) family receptor tyrosine kinase (RTK), to be highly expressed in the radioresistant tumour but not in the responsive tumour. Genetic deletion of Axl in the resistant tumours indicated that loss of Axl enhanced antigen presentation, altered cytokine secretion, and restored radiosensitivity that is largely immunologically mediated. Axl has gained much attention as it has been shown to be a key mediator of invasion and metastasis, is induced by HIF1-α and hypoxia, and can promote drug resistance when overexpressed^19^,^20^,^21^. In addition, Hugo *et al*.^22^ recently reported that Axl is a key gene expressed in patients unresponsive to PD-1 immunotherapy, which provides timely clinical relevance of our novel findings. Here we describe a previously unrecognized role for Axl in immune resistance that licenses an antitumour immune response when genetically targeted on the tumour cell. This antitumour response sensitizes tumours to RT and probably additional anticancer therapies.

## Results

### Differential radiosensitivity is not due to classic factors

**Figure 1:**
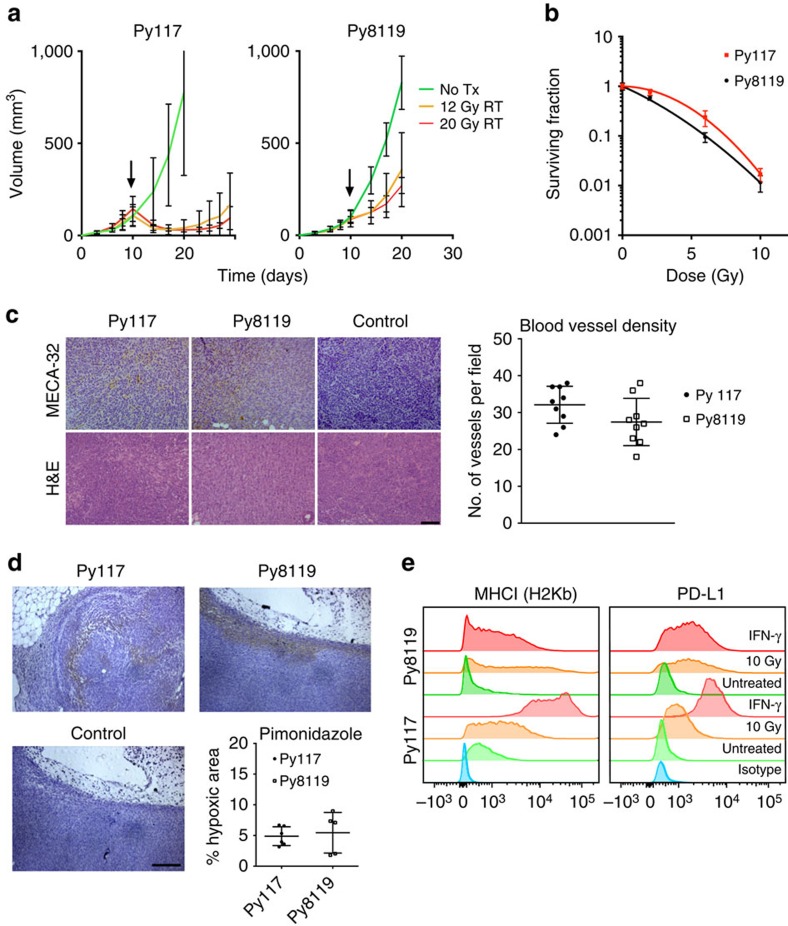
PyMT syngeneic tumours have different radiosensitivity that is not due to classic factors. (**a**) Orthotopic implantation reveals that Py117 tumours are more radiosensitive than Py8119 tumours and when untreated tumours have a similar growth rate (*n*=4–5 each group). (**b**) Clonogenic survival to evaluate intrinsic factors of radioresistance in culture showed no significant differences between the two tumour clones. (**c**) Blood vessel density was determined with MECA-32 staining showing no significant difference between two tumours (three mice, three fields per tumour; scale bar, 100 μm). (**d**) Levels of hypoxia are similar on the day radiation would be given as determined by pimonidazole immunohistochemistry (5–6 mice each; scale bar, 200 μm). (**e**) Immunological indicators, MHCI (H-2Kb) and PD-L1 were evaluated by flow cytometry. There was greater MHCI expression on the Py117 cells that was induced to a greater degree by 10 Gy or IFN-γ compared with Py8119 cells. PD-L1 was similar in the untreated cells but had greater induction by radiation in Py8119 cells whereas IFN-γ stimulated Py117 cells to a greater extent. All error bars, mean and s.d.

We developed a model of immunological heterogeneity in an isogenic background to study tumour-derived factors that influence immune mediated antitumour responses. Mammary tumour cell lines from the transgenic model of the mouse mammary tumour virus promoter driving the polyoma middle T antigen (MMTV-PyMT) in a C57Bl/6 background that efficiently form orthotopic tumours *in vivo* were used to evaluate radiobiological responses. The Py8119 clone was resistant to 12 and 20 Gy of radiation, whereas the Py117 clone was sensitive to these same doses (Fig. 1a). Both clones had similar radiosensitivity in cell culture as detected by clonogenic survival (Fig. 1b), indicating that tumour cell autonomous factors are not responsible for the differences in radiosensitivity. To determine if extrinsic factors of vascularization and hypoxia influenced the radiation response, we harvested tumours 90 min after injection of the hypoxia marker, pimonidazole (PIMO). Sections were stained with a MECA-32 antibody and anti-PIMO antibody to evaluate microvessel density and hypoxia, respectively. There were no significant differences between the Py117 and Py8119 tumours (Fig. 1c,d).

The Py117 and Py8119 cells were then evaluated for MHCI and PD-L1 surface expression to test the hypothesis that tumour cell immune mediated factors could be responsible for the differences in the radiation response. Although MHCI and PD-L1 were both enhanced by interferon gamma (IFN-γ), they have competing roles as MHCI promotes antigen-specific effector responses, while PD-L1 renders tumour cells resistant to T-cell effector functions. The Py117 cells had higher MHCI expression at baseline and after IFN-γ compared with Py8119 cells. In contrast, PD-L1 expression was low on both cell lines and was induced after radiation or IFN-γ treatment. IFN-γ mediated PD-L1 induction in both cell lines indicates that the adaptive immune resistance pathway is intact (Fig. 1e and Supplementary Fig. 1).

### Differential radiosensitivity is due to an immune response

**Figure 2:**
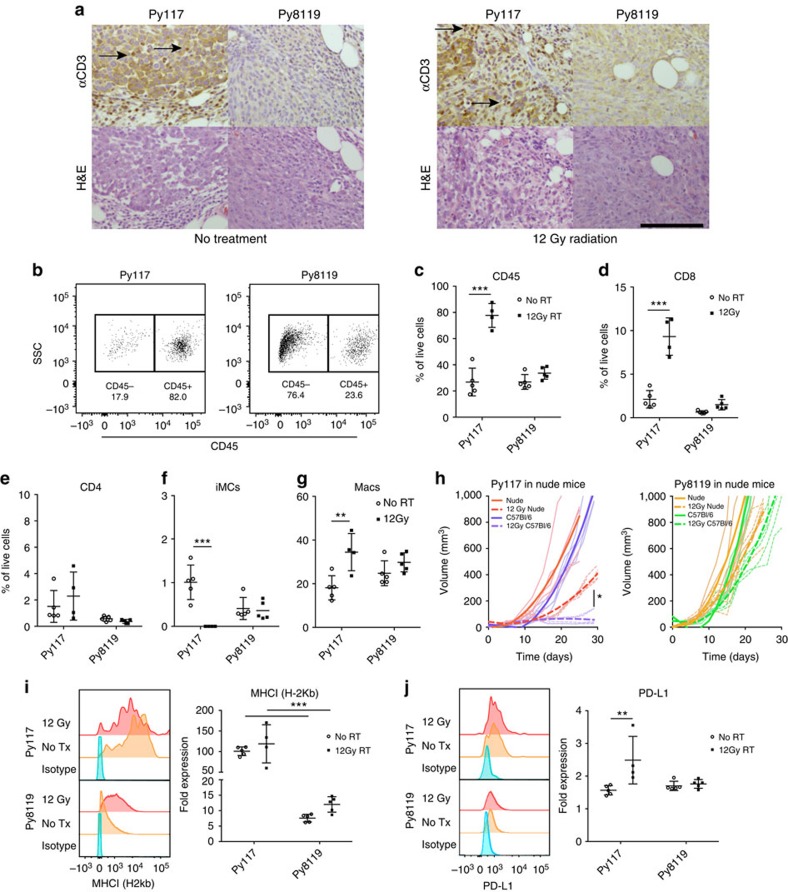
Radiosensitivity is associated with a differential antitumour immune response. (**a**) CD3 T-cell (arrows) immunohistochemistry 10 days after 12 Gy of radiation reveals increased infiltrates with and without radiation in Py117 compared with Py8119 tumours (scale bar, 100 μm). (**b**) Dissociated tumours analysed by flow cytometry had a greater proportion of CD45^+^ leukocytes after radiation in the Py117 tumours. (**c**) Quantification revealed an influx of leukocytes after radiation in Py117 tumours but not in Py8119 tumours (4–5 mice per group). The Py117 immune infiltrate consists of CD8^+^ T-cell influx (**d**), no change in CD4^+^ T-cells (**e**), a decrease in immature myeloid cells (iMCs: CD45^+^CD11b^+^Gr1^hi^) (**f**), and an increase in macrophages (CD45^+^CD11b^+^F4/80^+^) (**g**), with little differences in Py8119 tumours. (**h**) Implantation of tumours into nude mice revealed a decreased radiation response in Py117 tumours compared with syngeneic C57Bl/6 host and Py8119 curves were no different regardless of the host (Bold curves=repeated measures curve, thin curves=each mouse, five mice per condition). (**i**) MHCI (H-2Kb) is expressed on dissociated Py117 CD45^−^ cells at 10 × greater levels than in Py8119 tumours. (**j**) PD-L1 expression increased on dissociated Py117 CD45^−^ cells after radiation but not Py8119 cells. **P<0.007, ***P<0.0003 by two-way analysis of variance. *P=0.028 by repeated measures. All error bars, mean and s.d.

To evaluate differences in the immune response between Py117 and Py8119 cells, tumours were treated with or without 12 Gy of RT, and harvested after 10 days. Immunohistochemistry of CD3 revealed substantial numbers of T-cells in the untreated Py117 tumours that increased after RT. In contrast, there were few T-cells with or without RT in the Py8119 tumours (Fig. 2a). To quantify T-cell infiltrates and leukocyte populations, tumours were dissociated and analysed by flow cytometry. The percentage of CD45^+^ infiltrating leukocytes was similar in both untreated tumours, but increased from 27 to 78% 10 days after radiation in the Py117 tumours (Fig. 2b,c). The CD8^+^ T-cell subset increased from 2.1 to 9.3% of live cells. In addition, there were more CD4^+^ T-cells in Py117 compared with Py8119 tumours that did not change after radiation. There was almost a complete loss of immature myeloid cells often described as myeloid-derived suppressor cells or PMN-MDSCs with high GR1 expression (iMCs, CD45^+^CD11b^+^GR1^hi^)^23^ and lower proportion of macrophages (Macs, CD45^+^CD11b^+^F4/80^+^) that increased in Py117 tumours after radiation. In contrast, there was little change in these cell populations in the radioresistant Py8119 tumours (Fig. 2d–g, gating Supplementary Fig. 2).

To determine if the radiation response is dependent upon the immune system, tumours were implanted into athymic nude mice that lack functional T-cells. Py117 tumours grew faster in nude mice and were significantly less radiosensitive, confirming much of the sensitivity is due to a T-cell mediated immune response. The Py8119 tumours were equally radioresistant whether in a wild-type or immunodeficient host (Fig. 2h).

Given differences of MHCI and PD-L1 expression in cells in tissue culture, we investigated their levels on dissociated CD45^−^ tumour cells by flow cytometry. We found that MHCI was not significantly increased after radiation but was expressed at 10-fold greater levels in Py117 tumours than Py8119 tumours (Fig. 2i). Furthermore, a robust antitumour immune response in Py117 tumours after radiation was supported by increased PD-L1 expression, suggesting adaptive immune resistance. Unchanged levels of PD-L1 in Py8119 tumours after radiation support the lack of an interferon based immune response (Fig. 2j).

### Combination therapy is effective in radiosensitive tumours

**Figure 3:**
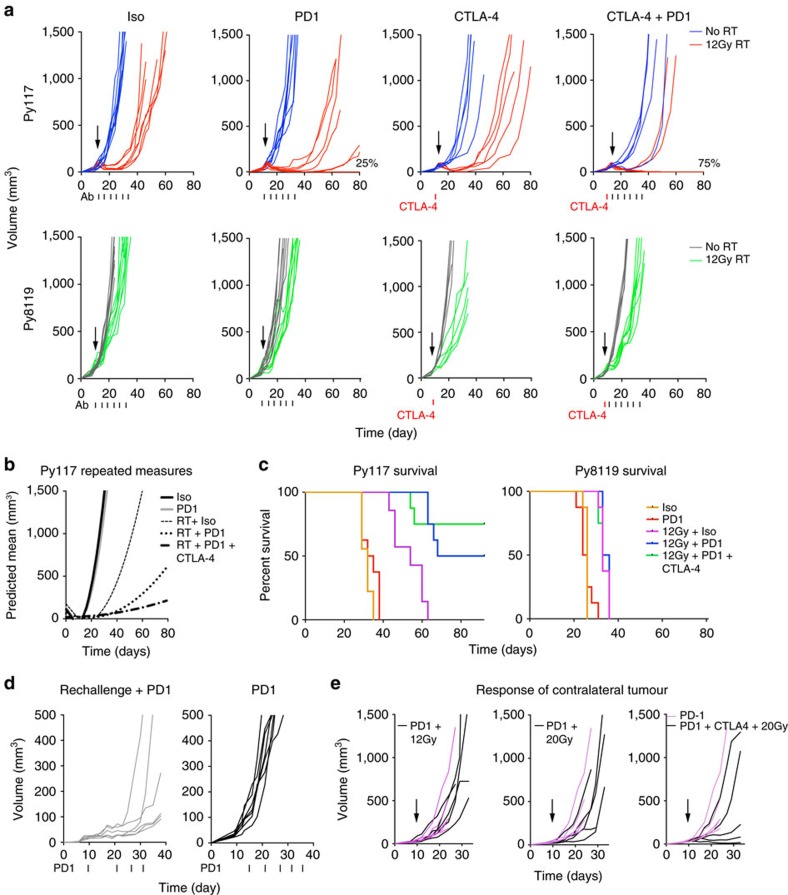
RT and immunotherapy leads to cures in the responsive Py117 tumours. (**a**) Tumour growth curves treated with and without 12 Gy RT plus isotype control Ab, PD-1 Ab, a single dose of CTLA-4 Ab 3 days before 12 Gy, or CTLA-4 and PD-1 Ab combination revealed cures with RT and PD-1 Ab in Py117 tumours but no response in Py8119 cells (%=per cent of eight mice with complete response, *n*=5–8). (**b**) The modelled Py117 tumour curves were significantly different between 12 Gy and 12 Gy+PD-1 or 12 Gy+PD-1+CTLA-4 (*P*=0.002 and 0.0004, respectively). (**c**) There was a difference in survival of Py117 but not Py8119 tumour bearing mice with radiation and PD-1 combinations (*P*<0.0001, log rank test). (**d**) Contralateral fat pad rechallenge in mice treated with 12 Gy+PD-1+CTLA-4 and PD-1 ab resulted in greater latency compared with PD-1 Ab alone. (**e**) Tumours were implanted in both inguinal fat pads and one tumour was radiated with 12 Gy or 20 Gy 10 days after implantation and combination of PD-1 or PD-1+ a single dose of CTLA-4 antibody revealing a response in the contralateral tumour in three of the five mice treated with 20 Gy+PD-1+CTLA-4 (five mice per group, purple curves=mice treated with PD-1 Ab alone).

With the finding of increased CD8^+^ T-cells, MHCI expression, and induction of PD-L1 after radiation in Py117 tumours we hypothesized that the combination of RT and PD-1 blockade would be highly effective in Py117 tumours, and be of little benefit in the Py8119 tumours. Mice were treated with 12 Gy of RT, Ab administration began on that day, and Ab was injected every 4 days for 24 days. Appropriate controls were included in the experimental scheme (Fig. 3a). Inhibition of PD-1 alone had little effect on Py117 tumours, but the combination of anti-PD-1 and RT led to a greater antitumour response, with 25% of the mice having no detectable tumour 80 days after treatment. To enhance T-cell priming and/or blocking regulatory T-cells (Tregs), a single dose of CTLA-4 Ab was given 3 days before the first RT and anti-PD-1 treatment, which led to a complete response in 75% of Py117 tumours. The effect of a single dose of CTLA-4 and its combination with radiation was similar to the PD-1 combination but the CTLA-4 and PD-1 combination resulted in more cures. The Py8119 tumours did not respond to a single agent or combination therapies (Fig. 3a). The Py117 tumour curves were modelled using a repeated measures model revealing a significant difference between 12 Gy+isotype versus 12 Gy+PD-1 Ab and 12 Gy+PD-1 Ab+CTLA-4 Ab with *P*=0.002 and 0.0004, respectively (Fig. 3b). The antitumour response of combination treatment lead to increased survival of mice with Py117 tumours but not mice with Py8119 tumours (Fig. 3c). Py117 mice were then treated with depleting antibodies for CD8^+^, CD4^+^ and NK cells to determine the relative importance of each cell type. We found that CD8^+^ depletion resulted in a diminished radiation response, depletion of NK cells had no effect, and depletion of CD4^+^ cells resulted in greater activity (Supplementary Fig. 3). This confirms the importance of CD8^+^ effector T-cells. Despite low number of CD4^+^ T-cells, if they are Tregs, they may blunt the antitumour response, explaining the added benefit of a single CTLA-4 dose^24^.

In preclinical studies of immunogenic tumour models, it is common to rechallenge cured mice and look at the response to an unirradiated contralateral tumour suggesting lasting immunity and ‘abscopal' responses^12^,^25^. To test if mice that had a complete response to combination therapy have lasting immunity, six out of the eight Py117 mice cured with 12 Gy+PD-1 Ab+CTLA-4 Ab were rechallenged in the contralateral fat pad. It was clear that all tumours were growing after 8 days, so PD-1 Ab was initiated. Though there was no rejection of the tumours, initiating PD-1 Ab was sufficient to slow tumour growth compared with naive mice treated with PD-1 Ab alone (Fig. 3d). To test for ‘abscopal' responses against a contralateral tumour, two tumours were implanted, one tumour received RT and the other tumour was monitored for growth delay. Mice were treated with 12 Gy or 20 Gy in a single fraction with PD-1 Ab or 20 Gy+PD-1+a single dose of CTLA-4 Ab 3 days before radiation. The group with a significant contralateral tumour response compared to the RT and PD-1 Ab treated mice was the combination of 20 Gy+PD-1 Ab+3 days pre-CTLA-4 Ab treatment (Fig. 3e). Therefore, Py117 tumours are responsive to combination therapy, but the response is not sufficient for complete eradication after rechallenge or for a complete response to an unirradiated contralateral tumour. These data suggest that radiation plays a key role in licensing an immune response that can be enhanced with checkpoint blockade, but is not sufficient for a complete long-term antitumour immune response, which may be due to the absence of a dominant tumour antigen. Radiation does not reverse T-cell infiltration in the Py8119 tumours, suggesting a tumour cell intrinsic mechanism of T-cell exclusion may play an important role.

Many of the models which describe robust antitumour immune responses, efficient abscopal responses, and resistance to rechallenge, introduce model antigens such as HA, OVA, or SIY peptides^12^,^13^. We did not perform genetic manipulation of the cells, but we performed whole exome sequencing of the Py117 and Py8119 cells to ensure there was no dramatic difference in mutation burden. Focusing on mutations unique to each cell line, we found that the Py117 cells contained 83 unique mutations resulting in stop-gain, frameshift or nonsynonymous variants whereas the Py8119 cells contained 66 (Supplementary Data 1). These data suggests that differences in mutation burden between the two cell lines are unlikely to explain the difference in the immune response we observed. Kim *et al*. recently sequenced the poorly immunogenic syngeneic 4T1 breast cancer cell line that contained 47 mutations and the moderately immunogenic CT26 colorectal cancer cell line that contained 683 mutations and despite difference in mapped epitopes on MHCI there was little difference in the immune response generated. They found that pharmacological treatment with epigenetic modulators did not impact antigen presentation as much as they had effects on suppressive myeloid cells, despite a 10-fold difference in mutations between the models^17^. We hypothesize that the ten-fold difference in MHCI expression (Fig. 2i) or other factors are more likely to impact the immune response than the mutational burden.

### Axl impacts the immune response and tumour growth

**Figure 4:**
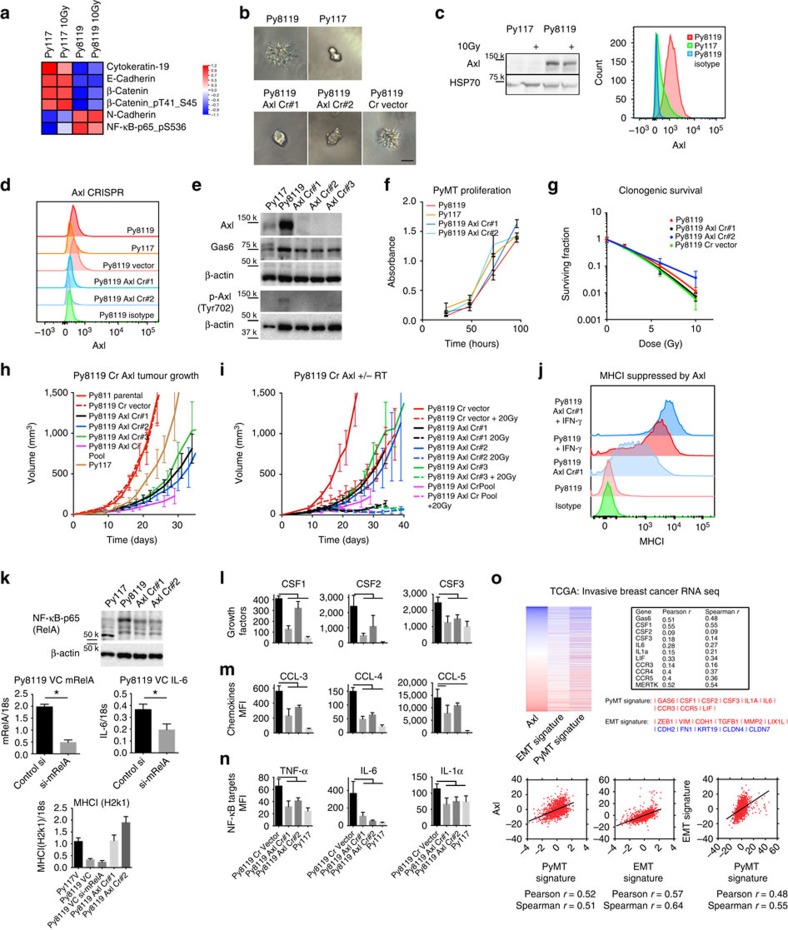
Axl impacts key immunosuppresive immunological factors in tumour cells. (**a**) The Py8119 cells have an EMT pattern on RPPA and (**b**) are more invasive than Py117 cells in 3D culture (scale bar, 50 μm). (**c**) There was elevated Axl compared with Py117 cells with or without 10 Gy of radiation on western blot (left), and flow cytometry (right). (**d**) CRISPR knockout of Axl (Py8119 Axl Cr#1/ #2) was confirmed by flow cytometry and loss of Axl resulted in decreased invasion in 3D culture (**b**). (**e**) Western blots confirmed Axl knockout and decreased phospho-signaling in three CRISPR clones. (**f**) There was no difference in proliferation between wild-type and Axl Cr clones (XTT assay, representative experiment, in triplicate) and (**g**) clonogenic survival revealed no difference in intrinsic factors of radiosensitivity (three independent experiments). There was delayed growth (**h**) and enhanced radiosensitivity (**i**) of Axl knockout tumours (three independent clones and pooled) compared with parental, vector control, and Py117 tumours (five mice each, RT: Py8119=day 9, Axl Cr#1–3=day 14, and Axl Cr Pool=day 19). (**j**) MHCI surface expression was increased in Axl Cr #1 cells and further induced by IFN-γ compared with parental cells. (**k**) NF-κB-p65 protein was decreased in Axl Cr #1 and #2 and siRNA knockdown in Py8119 vector control (VC) cells revealed knockdown of the transcript and NF-κB target IL-6 (triplicate, *P<0.03 *t*-test, MHCI ANOVA *P*<0.0001). Conditioned media from Py8119 VC, Axl Cr#1/#2, and Py117 cells resulted in decreased myeloid supporting growth factors (**l**), myeloid recruiting chemokines (**m**), and NF-κB targets (**n**) (MFI=mean fluorescence intensity; three independent experiments; analysis of variance *P*<0.05 for each molecule, *P*<0.03 for pairwise comparison with Py8119 VC. (**o**) RNAseq from the cancer genome atlas breast cancer data set (*n*=1215) revealed a correlation between Axl and proteins decreased in the Axl knockout cells. A PyMT and EMT gene signature was derived that revealed a significant correlation with Axl and each signature (red=positive and blue=negative correlation, all Pearson and Spearman *P*<0.0001 except for CSF2 that were 0.0024 and 0.024, respectively). All error bars, mean and s.d.

To determine differences in signalling pathways between the tumour clones, we used reverse phase protein arrays (RPPA). The analysis resulted in a number of differences, supporting an independent clonal evolution of the two cell lines as expected. The Py8119 tumour cells had clear differences in epithelial mesenchymal transition (EMT) -related proteins including reduced E-Cadherin, phospho β-catenin, and cytokeratin, and increased N-Cadherin and phospho NF-κB (Fig. 4a). This observation is consistent with differences in cell invasion in three-dimensional (3D) culture in Fig. 4b. We found that phospho β-catenin is reduced in Py8119 compared with Py117 cells, suggesting it is not the key mediator of immune suppression in this model as has been reported in melanoma^16^.

Consistent with changes in the EMT phenotype and cell morphology, the RTK Axl was elevated on Py8119 compared with Py117 cells. Western blot and cell surface staining by flow cytometry confirmed elevated expression in the resistant Py8119 cells compared with the Py117 cells, and that the expression of Axl did not change with radiation (Fig. 4c). We hypothesized that Axl has a role in immune resistance given its role in EMT, the observation that Axl is differentially expressed between the two tumour cell lines, and how it could impact myeloid cells in suppressing an anti-tumour immune response if there was an increase in subsequent Gas6 expression^26^,^27^,^28^. To better understand the immunological role of Axl, the CRISPR/Cas9 technology was used to knockout Axl in Py8119 cells. Figure 4d shows untreated and Supplementary Fig. 4A shows IFN-γ treated cells changes in Axl expression by flow cytometry in wild-type Py8119 cells, vector control Py8119 cells, Py117 cells and five Py8119 Axl CRISPR tumour cell clones (Axl Cr#1–5). Western blot analysis confirmed elevated expression of Axl protein in Py8119 cells as well as higher levels of Tyr702 phosphorylation. In contrast, complete knockout of Axl in the CRISPR clones resulted in undetectable levels of Axl protein and Tyr702 phosphorylation (Fig. 4e).

The Axl CRISPR knockout Py8119 cells showed a loss of invasive capacity in 3D culture (Fig. 4b and Supplementary Fig. 4B) but did not show an impact on proliferation rate in tissue culture (Fig. 4f). There was no difference in *in vitro* radio-sensitivity based upon clonogenic survival of the knockout clones when compared with parental cells from Fig. 1b (Fig. 4g). When the CRISPR cells were implanted into naive C57Bl/6 mice, there was slower tumour growth in three clones and pooled clones compared with CRISPR control and parental Py8119 tumours (Fig. 4h). Lastly, we found that Axl knockout impacts radiosensitivity in the three different Axl knockout clonal tumours in the context of the tumour microenvironment and immune system but not in Py8119 vector control orthotopic tumours (Fig. 4i).

### Axl suppresses MHCI and enhances cytokine secretion

Given low MHCI levels in Py8119 cells compared with Py117 discussed in Fig. 1, we hypothesized that Axl suppresses MHCI expression. Rothlin *et al*.^26^ observed increased MHCI expression in myeloid cells of TAM triple knockout mice, suggesting an association. We found that MHCI surface expression was increased in Axl knockout clones Axl Cr #1 and #2 compared with control Py8119 cells (Fig. 4j and Supplementary Fig. 5A). We investigated the MHC transcriptional co-activator CIITA that can be influenced by EGFR inhibition but found no enhancement of CIITA transcription, although levels were higher in Py117 cells^29^ (Supplementary Fig. 5B). We found increased STAT1 protein and phosphorylation in Py8119 Axl CRISPR cells, a known mediator of IFN-γ and MHCI expression, which suggests that Axl directly or indirectly suppresses STAT1 (Supplementary Fig. 5C). However, Axl knockout cells did not result in an increase in PD-L1 expression in the absence of IFN-γ stimulation (Supplementary Fig. 5D).

We also tested the hypothesis that NF-κB targets in the tumour cells may be differentially activated given the elevated activity observed in the RPPA. Increased protein levels of NF-κB p65 were found in wild-type compared with knockout cells. NF-κB-p65 was targeted with siRNA, which resulted in decreased IL-6 transcription compared with Py8119 vector control cells. MHCI levels were not affected in NF-κB siRNA cells, indicating that its suppression by Axl is not NF-κB-dependent (Fig. 4k).

Elevated levels of Axl may not only suppress MHCI but could promote myeloid cells such as macrophages through secretion of soluble factors. The Luminex multiplex cytokine and chemokine assay was performed on conditioned media from vector control Py8119 cells, two Py8119 Axl Cr clones, and wild-type Py117 cells, revealing a number of factors significantly decreased in Axl knockout and Py117 cells. The factors identified fall into three categories: myeloid supporting cytokines (CSF1, CSF2 and CSF3) (Fig. 4l); macrophage recruiting chemokine's (CCL3, CCL4 and CCL5) (Fig. 4m), and NF-κB target cytokines (IL-6, TNF-α and IL-1α) (Fig. 4n).

These findings suggest that the Py8119 cells amplify TAM signalling through Gas6 production, enhanced NF-κB inflammatory response including cytokine targets, may recruit myeloid cells through cytokine and chemokine secretion, and suppress antigen presentation through MHCI. Taken together, we hypothesized that Axl is a key suppressor of an anti-tumour immune response. Though Axl has been known to mediate therapeutic resistance, the recent study by Hugo *et al*.^22^ was the first to suggest a role in resistance to immunotherapy. To evaluate if our findings correlate with gene expression in human cancer, we investigated the cancer genome atlas (TCGA)^30^. In the breast cancer data set of 1,215 patients, Axl significantly correlates with many of the secreted factors identified on Luminex in addition to Axl associated genes Gas6 and Mer. We generated a gene signature (PyMT Signature) that correlated well with Axl expression (Pearson *r* value of 0.52). Because Axl is important in EMT, we generated an EMT signature adapted from Byers *et al*.^31^ and found a significant relationship to Axl expression and our PyMT signature, suggesting that the factors secreted in the presence of Axl signalling are EMT related (Fig. 4o). We then checked the PyMT signature against Axl expression in the PanCancer data set of 9,755 patients, subdivided the different disease sites, and evaluated the Cancer Cell Line Encyclopedia of 967 human cancer cell lines, and found significant correlations (Supplementary Fig. 6).

### Axl suppresses the antitumour immune responses

**Figure 5:**
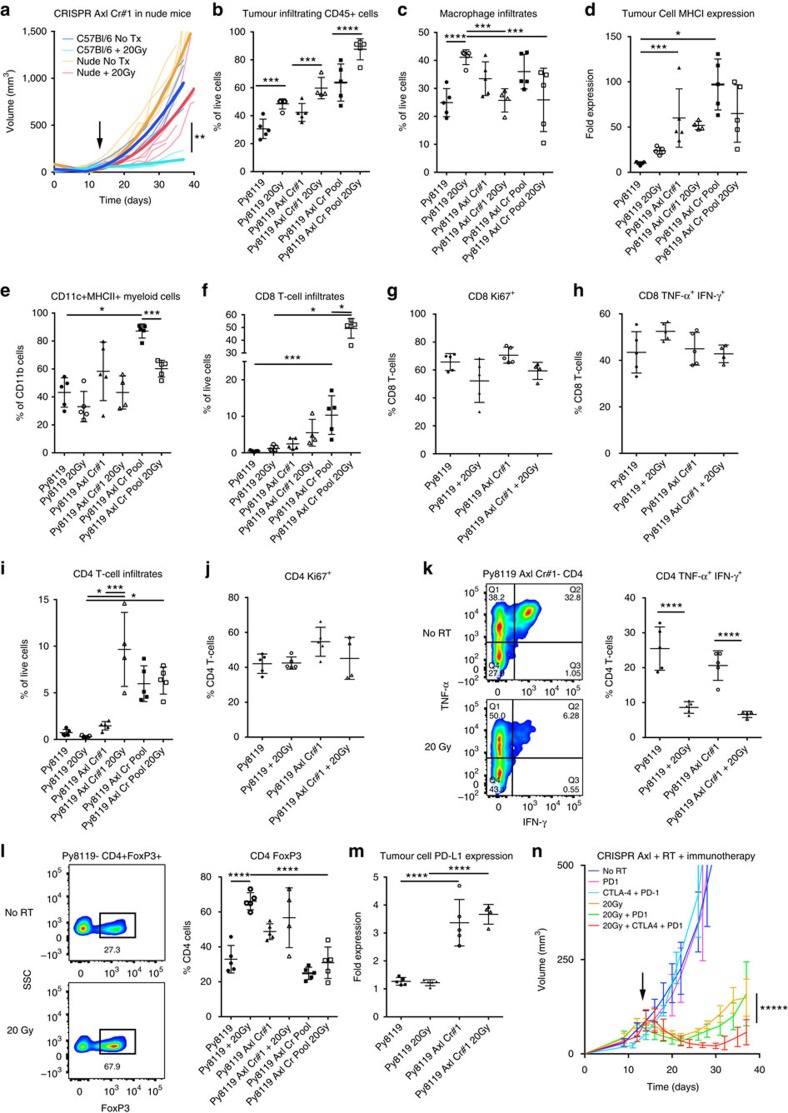
Adaptive immune response contributes to Axl mediated radiosensitivity. (**a**) The radiation response of Py8119 Axl Cr#1 to 20 Gy is decreased in immunodeficient nude mice (RT at 12–14 days, **P=0.0036, five mice each). (**b**) There was an increased proportion of infiltrating CD45^+^ leukocytes in Axl Cr#1 and pooled tumours and in all tumours 10 days after 20 Gy. (**c**) The proportion of CD45^+^CD11b^+^F4/80^+^ macrophages increased after radiation in Py8119 tumours but was higher initially then decreased in Axl Cr#1 tumours. (**d**) MHCI expression was greater in Axl knockout CD45^−^ tumour cells compared with parental tumours. (**e**) Despite a decrease in macrophages, the proportion of antigen presenting CD11b^+^CD11c^+^MHCII^+^ cells was greater in Axl deficient tumours with and without radiation. (**f**) There were greater infiltrating CD8^+^ T-cells in Axl knockout tumours that increased after radiation. (**g**) The CD8^+^ cells had an elevated proportion of Ki-67^+^ cells in wild-type and Axl Cr#1 tumours. (**h**) The CD8^+^ T-cells were isolated and stimulated revealing a significant proportion of cells with capacity to secrete TNF-α and IFN-γ. (**i**) CD4^+^ T-cell infiltrates decreased after radiation in wild-type tumours where the knockout tumours had a higher baseline that increased after RT. (**j**) The proportion of Ki-67 CD4^+^ cells was unchanged in wild-type and Axl knockout tumours. (**k**) The proportion of TH-1-like TNF-α and IFN-γ producing cells decreased in irradiated tumours. (**l**) The CD4^+^FoxP3^+^ Treg proportion increased in Py8119 tumours after radiation. The less responsive Axl Cr#1 compared with the pooled tumours had a greater proportion of Tregs at baseline and neither was impacted by RT. (**m**) PD-L1 expression was higher on Axl Cr#1 tumour cells suggesting adaptive resistance in the setting of a greater inflammatory response (4–5 mice each; *P<0.05; ***P<0.01; ****P<0.001 by two-way ANOVA). (**n**) Given elevated Tregs and PD-L1 in Axl Cr#1 tumours, CTLA-4 and PD-1 Ab was combined with 20 Gy resulting in a greater therapeutic response (arrow=RT, *****P=0.0006 for RT vs RT+PD1 Ab+CTLA-4 Ab, five mice each). All error bars, mean and s.d.

We aimed to evaluate the impact of Axl knockout tumours on the immunological microenvironment to determine if loss of Axl licenses an antitumour immune response. Py8119 Axl knockout clones were injected into nude mice and irradiated when tumours were 75–125 mm^3^ to determine if immunodeficiency impacts radiation response. Tumours derived from both Axl Cr#1 and 2 knockout cells had reduced response to radiation when implanted in nude mice compared with wild-type mice, with the time to doubling volume of 13 days in nude mice versus 25 days in wild-type mice (Fig. 5a and Supplementary Fig. 7). Py8119 Axl Cr#1 or pooled knockout tumours from untreated or irradiated groups were dissociated 10 days after treatment and compared with the parental Py8119 tumours. The Axl knockout tumours had a greater proportion of CD45^+^ infiltrates compared with parental tumours without irradiation, which increased after radiation, supporting that loss of Axl results in a greater inflammatory phenotype (Fig. 5b). Macrophage infiltration was greater in Axl knockout tumours at baseline given a higher proportion of CD45^+^ cells. However, the proportion of macrophages in Axl knockout tumours significantly decreased after radiation compared with an increase in parental tumours (Fig. 5c). Thus the loss of Axl results in decreased levels of macrophages in response to radiation and suggests an important role of Axl in the recruitment of macrophages after radiation, which supports the cytokine and chemokine signalling hypothesis.

After finding increased immune infiltrates in Axl knockout tumours, we hypothesized that the loss of Axl elicits a T-cell mediated immune response through enhanced tumour cell and myeloid cell antigen presentation. As observed in tissue culture, the tumour-derived CD45^−^ cells exhibited increased surface expression of MHCI 50–100 fold after the knockout of Axl with and without radiation (Fig. 5d). With greater antigen presentation and CD45^+^ infiltrates, we evaluated the proportion of CD11b^+^ antigen presenting myeloid cells that express CD11c and MHCII, often considered myeloid dendritic cells (mDCs: CD45^+^CD11b^+^CD11c^+^MHCII^+^)^32^. We found greater proportion of mDCs in the Axl knockout tumours (Fig. 5e and Supplementary Fig. 8). However, after radiation the mDCs are relatively decreased perhaps due to T-cell influx, diminished myeloid cell recruitment, and a robust Treg response that can suppress mDCs and promote M2-like macrophages^33^,^34^.

In addition to antigen presentation and mDC infiltrates there was also an increase in CD8^+^ T-cells without treatment compared with wild-type tumours, and T-cell infiltrates increased even more after radiation. The fold increases in proportion of CD8^+^ cells in the Py8119 Axl Cr#1 and Py8119 pool compared with wild-type tumours were 4.9 and 20.6 without RT and 4.5 and 40.7 after RT (Fig. 5f). The CD8^+^ T-cells had a high proportion of the Ki-67 proliferation marker in all groups (Fig. 5g). Upon stimulation, the CD8^+^ T-cells produced TNF-α and IFN-γ supporting a functional phenotype rather than exhausted T-cell phenotype (Fig. 5h, Supplementary Fig. 9A,B). After initial regression, tumour progression occurred, suggesting that either exhaustion or adaptive immune resistance occurs despite an antitumour response. The highest proportion of PD-1^+^ cells was observed in the wild-type untreated tumours and was lower after radiation and in Axl knockout tumours. These data support that a significant proportion of T-cells may become functionally inactive upon engagement with the PD-L1 ligand (Supplementary Fig. 10A).

We next evaluated the impact of Axl on CD4^+^ T-cells to determine if these cells support a TH-1 antitumour phenotype or whether they augment the antitumour response through Treg recruitment. Surprisingly, CD4^+^ cells were at a low abundance in wild-type Py8119 tumours and further decreased 10 days after radiation. The number of CD4^+^ cells was initially higher then increased after radiation in the Axl Cr#1 tumours. In comparison, CD4^+^ cells were high with or without radiation in the responsive pooled knockout tumours (Fig. 5i). The proportion of CD4^+^ cells that secrete TNF-α and IFN-γ upon stimulation decreased after radiation in both tumours suggesting a decreased TH-1 response (Fig. 5k and Supplementary Fig. 9C). The abundance of FoxP3^+^ Treg cells increased in wild-type tumours after radiation. Alternatively, there was no relative increase in Tregs in knockout tumours. Tregs were abundant after radiation but more so in Axl Cr#1 compared with the pooled tumours given the increased CD4^+^ infiltrates (Fig. 5l). The increase in FoxP3^+^ Tregs in the Axl knockout tumours suggests an adaptive immune resistance mechanism after radiation that is greater in the less responsive Axl Cr#1 tumours compared with the pooled Axl CRISPR tumours. This mechanism may explain why tumours recur rather than resolve despite the robust CD8^+^ T-cell response. Evaluation of PD-L1 levels in CD45^−^ tumour cells indicated the PD-1/PD-L1 pathway may also be a second mechanism of adaptive resistance (Fig. 5m). Overall the pooled Axl CRISPR tumours had the slowest growth and were the most sensitive to radiation, suggesting a greater immune response than the single Axl Cr #1 clone, which could be explained by greater CD45^+^ cells, mDCs, CD8^+^ cells, CD4^+^ cells and relatively fewer Tregs. Furthermore, improved overall antitumour T-cell response in the pooled Axl CRISPR tumours is also supported by an enhanced CD8^+^ to Treg ratio (Supplementary Fig. 10B).

Because of elevated Tregs and PD-L1 expression, we hypothesized that targeting Tregs with CTLA-4 and PD-L1 with a PD-1 blocking antibody would lead to a greater antitumour immune response after radiation. PD-1 antibody alone had little effect on tumour growth, and the combination of PD-1 Ab and 20 Gy was no better than radiation alone. However, the combination of CTLA-4 Ab, PD-1 Ab, and radiation resulted in a greater response than 20 Gy and 20 Gy+PD-1 Ab (Fig. 5n). There was little to no response to this combination therapy in wild-type Py8119 tumours (Supplementary Fig. 11).

## Discussion

**Figure 6:**
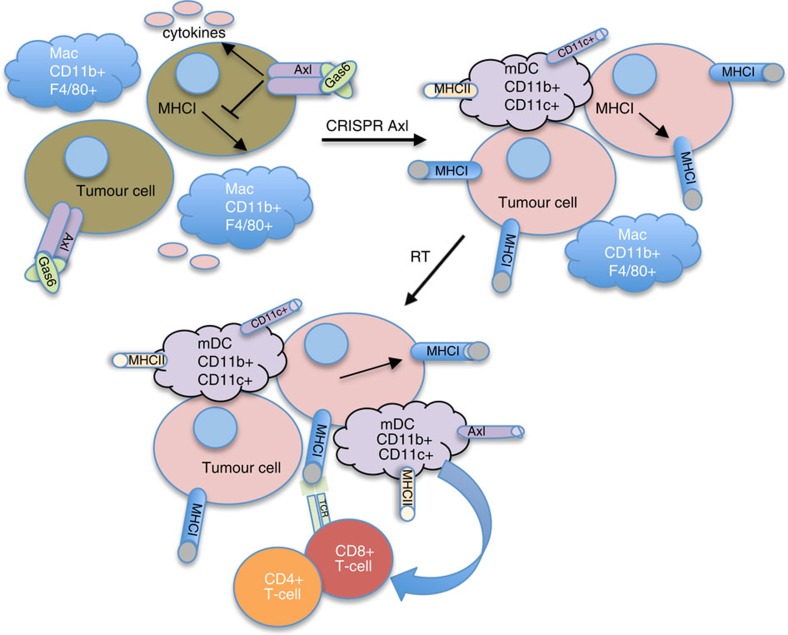
Loss of Axl enables licensing an antitumour immune response after RT. With high expression of Axl, there is low MHCI expression and tumour cells release myeloid promoting cytokines and chemokines. With the loss of Axl, MHCI expression increases and cytokine signaling is diminished leading to an immune response with increased CD11c^+^ and MHCII^+^ antigen presenting cells (mDC) and T-cells. The addition of RT licenses a robust antitumour immune response with decreased macrophages (Mac), increased proportion of antigen presenting cells, and a greater recruitment of CD4^+^ and CD8^+^ T-cells.

With the expanding role of immune checkpoint therapies and their clinical impact, much attention has been focused on the immune system and its potential to eradicate cancer. However, there is a need to understand how tumours evade an immune response, often rendering checkpoint therapies ineffective. In this study, we used tumour clones from a transgenic mouse model to discover factors that contribute to immune resistance after radiation. Figure 6 depicts our current understanding of how tumour Axl impacts the immune response. Genetic deletion of Axl in the resistant tumour cells leads to an increase in MHCI and myeloid promoting cytokines. Additionally, these key molecular findings correlate with Axl expression in the TCGA breast cancer data set, and our molecular signature correlates with Axl and an EMT gene signature. In this model system, loss of Axl allows the development of antitumour immune response where it was previously suppressed. RT results in greater CD45^+^, mDC, CD8^+^ cells, but decreased macrophage infiltration. The immune response generated by loss of Axl leads to adaptive immune resistance through PD-L1 expression and Treg infiltration and tumours became sensitive to checkpoint immunotherapy when they were previously resistant.

Our findings provide complementary insight to Hugo *et al*., who showed that Axl and other EMT genes are correlated with resistance to PD-1 immunotherapy in melanoma patients^22^. We propose that in some tumours anti-Axl therapy will enhance antitumour immune responses to overcome therapeutic resistance, while at the same time decrease invasion, and metastasis. Therefore, RT or other conventional therapies may optimally combine with Axl inhibition in order generate an immune response that may otherwise be suppressed.

In recent years, Axl has been identified as a therapeutic target for its role in cancer progression. Multiple therapeutics directed against Axl are in development including kinase inhibitors, soluble receptors, and antibodies^35^,^36^,^37^,^38^. There is growing recognition that TAM family RTKs, including Axl and Mer play important roles not only in invasion and metastasis and therapeutic resistance but also in the generation of an anti-inflammatory immunosuppressive environment. Axl has suppressive effects through the inhibition of dendritic cell activation and along with Mer can decrease NK cell antitumour activity resulting in increased metastasis^26^,^39^,^40^. In addition, Mer is important in efferocytosis or clearance of dead cells by phagocytosis, suppression of macrophage cytokine responses, promoting an M2 macrophage phenotype, promoting B-cell tolerance, and T-cell mediated suppression of antigen-presenting cells^21^.

MHCI expression was one of the biggest differences between the PyMT tumour cell clones. Even with IFN-γ stimulation, MHCI levels could not be restored in Py8119 cells to the levels observed in the Py117 cells (Fig. 1e). The fact that invading cells with an EMT phenotype like Py8119 need to escape immune surveillance, and kinases such as EGFR have been implicated in suppressing MHCI, we hypothesized that Axl may suppress MHCI^29^. CRISPR knockouts of Axl resulted in an increase in MHCI that could be induced to levels higher than IFN-γ stimulated parental cells (Fig. 4k). The effects impact both STAT1 translation and phosphorylation. Therefore, Axl seems to play a role in regulating antigen presentation and studies are ongoing to better understand this observation.

The role of NF-κB activation and induction of inflammatory cytokines likely contributes to a tumour promoting microenvironment. Both TNF-α and IL-6 have been previously reported to be associated with TAM RTK signalling^41^,^42^. Here we found that both TNF-α and IL-6 production by tumour cells is diminished upon Axl knockout. In addition, we found a significant decrease in CSF1, CSF2, CSF3, CCL3, CCL4, CCL5 and IL1-α in Axl knockout cells. These cytokines and chemokines each play a role in supporting myeloid cell infiltration in the tumour microenvironment and may explain high levels of macrophages that increase after radiation. In Axl knockout tumours, the macrophages decreased after radiation despite an absolute increase in leukocytes before therapy (Fig. 5b). In addition, there was an increase in the number of myeloid dendritic cells that may prime T-cells and in irradiated tumours result in T-cell infiltration and immune dependent radiosensitivity.

This study reinforces the concept that immune checkpoint therapies are not effective unless an antitumour immune response is generated. The Py117 tumour cells have high levels of MHCI expression and infiltrating T-cells at baseline, but established Py117 tumours are not responsive to PD-1 therapy alone. This unresponsiveness may be due to the lack of dominant antigens to activate a robust T-cell response. We hypothesize that radiation serves as a catalyst to remove suppressive factors, release tumour antigens, and kill tumour cells enabling an adaptive immune response. In this setting, anti-PD1 therapy provides additional benefit to block adaptive resistance. In the Axl expressing Py8119 tumours, we observed a limited initial immune response, which was not improved by radiation, thereby making combination with checkpoint therapy ineffective. Therefore, Axl directed therapy in Axl expressing tumours could reprogram resistant tumours when combined with radiation or chemotherapy and immunotherapy. These findings have significant clinical implications, and it will be important to more completely elucidate the major effects of Axl inhibition in various contexts as we begin to propose anti-Axl therapy in combination with immunotherapies.

## Methods

### PyMT tumour cell lines

Spontaneous tumours from the transgenic MMTV-PyMT mice congenic in the C57Bl/6 background^43^ were enzymatically digested for two to three hours at 37 °C in 1 mg ml^−1^ type 2 collagenase (Worthington Biochemical Corp.), 2 mg ml^−1^ soybean trypsin inhibitor (Sigma-Aldrich), 1 mg ml^−1^ BSA (Sigma-Aldrich, MO), 50 mg ml^−1^ gentamicin (Life Technologies) in F12K media (Mediatech Inc.). The suspension was neutralized in serum containing media and filtered through 70 μm mesh, centrifuged and resuspended in complete F12K media containing 5% fetal clone II (Hyclone), MITO (1:1,000 dilution, BD Biosciences), 50 μg ml^−1^ gentamicin and 2.5 μg ml^−1^ amphotericin B as previously described^44^. The cells were passaged for approximately five passages and cloned by limiting dilution. Clonal cell lines were maintained in complete F12K media. There was no other selection difference during the derivation of Py8119 and Py117 cells such as selection of drug resistant clones. All experiments were performed with Py8119 and Py117 clones between passage numbers 15–25. Cells were confirmed to be mycoplasma free.

For tissue culture experiments cells were treated as described in the text. For radiation and clonogenic survival cells were irradiated and then plated accordingly such as limiting dilutions for colony formation. For cells plated for 3D culture, 2,500 cells were plated in 2 mg ml^−1^ low growth factor matrigel (Corning) in 96-well plates after a base coat was established. Media was changed daily and cell clusters were imaged by phase contrast at 4 days. For syngeneic orthotopic implants 1 × 10^6^ tumour cells were injected into the mammary fat pad in 2 mg ml^−1^ matrigel (Corning). For XTT assay cells were plated at 2–5,000 cells per well in 96-well plate and treated in triplicate groups of cells at 24, 49, 72 and 96 days after plating. Cells were analysed on a plate reader using absorbance based off of XTT assay recommendations.

### Tumour treatments

RT of tumours was typically initiated between 75 and 125 mm^3^ on day 9–14 after implantation (the pooled CRISPR tumours were treated on day 19 due to slow growth). Each cell line and model were treated on the same day, however, the nude and C57Bl/6 comparison growth curves of the Axl Cr#1 tumours were adjusted to the date RT was administered while in the target size range given the discrepancy in growth rate. Volume measurements were obtained every 2–4 days after initiation of treatment using *L* × *L* × *W*/2. RT was performed using a 225 kVp cabinet X-ray irradiator filtered with 0.5 mm Cu (IC-250, Kimtron Inc., CT) and anaesthetised animals were shielded with a 3.2 mm lead shield with a 15 × 20 mm aperture. CTLA-4 Ab (9D9, Bio-X-Cell) treatment using 200 μg IP injection was administered 3 days before the day of RT or as described in the text and then every 4 days × 5 in the combination therapy Axl knockout tumour experiment. PD-1 Ab (RMP1-14, Bio-X-Cell) treatment with 200 μg IP was initiated on day of radiation and administered every 4 days × 5–6 (five doses given if the tumour diameter was near 17.5 mm). For blocking antibody experiments αCD8 Ab (2.43, Bio-X-Cell), αCD4 (GK1.5, Bio-X-Cell), αNK1.1 (PK136) or Rat IgG2a isotype Ab were injected IP at 500 μg per injection 1 day before radiation (day 13) then every 5 days × 4 total. For the Axl knockout radiation and immunotherapy experiments radiation was performed 14 days after implantation and CTLA-4 was injected IP 3 days before radiation then injected with PD-1 at the time of radiation and every 4 days × 5. All animal procedures were approved by the administrative panel on laboratory animal care.

### Immunohistochemistry

Tumour-bearing mice were injected with 15 mg of Pimonidazole (Pimo, Hypoxyprobe-1 Omni Kit, 1:150, PAb2627AP, Hypoxyprobe Inc., MA) IP 90 min before harvest on the day radiation would be given. Tumours were removed, fixed in 10% formalin buffered saline and embedded in paraffin for histology and IHC. Tumour sections were dewaxed, citric acid antigen retrieval was performed, and sections were stained using a standard HRP/DAB kit (Vector laboratories, CA). Rat anti-mouse panendothelial cell antigen (MECA-32, 1:250, 550563, BD Biosciences) and rabbit anti-Pimo staining was performed to determine baseline tumour characteristics before treatment when tumours were ∼100 mm^3^. For anti-CD3 staining, Py8119 and Py117 tumours from a cohort of mice that were untreated or treated with 12 Gy RT when average tumour size was 100 mm^3^ were harvested 10 days after treatment and paraffin embedded. Tumour sections were treated as above and stained with rabbit polyclonal anti-CD3 antibody (1:200, AB5690, Abcam). DAB was developed until precipitation was noted in specific areas of tumour sections. In all cases control staining's with no primary antibody was performed to confirm specific HRP-DAB precipitation. Serial sections of hematoxylin and eosin (H&E) were obtained. Slides were imaged using a Leica DM6000B microscope (Leica, Germany). MECA-32 microvessel staining quantification was performed using the Photoshop counting tool evaluating three tumours and three fields per tumour at × 200 magnification. Pimo was quantified using ImageJ by imaging multiple tumour sections at × 100 integrating total hypoxic area divided by the total tumour area at to determine the % hypoxic area per tumour.

### Flow cytometry

For cell experiments, tumour cells grown *in vitro* trypsinized, washed, stained with fluorophore labelled antibodies for 20 min on ice in PBS containing 3% FBS staining buffer, washed two times in × 10 volume staining buffer and kept on ice then analysed by flow cytometry. For IFN-γ treatments 3.33 × 10^5^ cells were treated with 25 ng ml^−1^ of mouse recombinant IFN-γ (Peprotech) overnight in serum containing growth media. For tissue culture radiation experiments, 1 × 10^6^ cells were resuspended in 1 ml of growth media, irradiated using a Cs source and then plated at 3.33 × 10^5^ cells in six well plate for 24 h before harvesting for flow cytometry.

To create cell suspensions, tumours were removed, finely chopped, and suspended in 1:1 F12K media and DMEM 5% FBS. Tumours were digested with collagenase type I at 200 U ml^−1^ (Worthington) and Dispase at 0.5 U ml^−1^ (Stem Cell Technologies, Canada) for 40 min at 37 °C then filtered through a 40 μm mesh. Cells were resuspended in RBC lysis buffer for 10 min at room temperature. Cells were resuspended in PBS, counted and then stained with Zombie NIR (BioLegend) for live/dead cell discrimination. Cells were washed, fixed with 5% formalin buffered saline for 20 min on ice washed, and then frozen at −80 °C in cell staining buffer until flow cytometry was performed. On the day of analysis cells were thawed on ice, FC receptors were blocked with CD16/32 Ab (BioLegend), and then 1 × 10^6^ were stained with conjugated Ab cocktail for 20 min on ice. Cells were washed 2 × then resuspended for flow cytometry analysis.

For T-cell function experiments tumours were digested with 20 μg ml^−1^ Liberase TL (Roche) and 400 μg ml^−1^ DNAse (Roche) then digested for 30 min followed by Percoll (VWR) centrifugation for larger tumours. Cells were stimulated with 50 ng ml^−1^ of PMA and 500 ng ml^−1^ Inomycin for 3 h in the presence of 1x Brefeldin A (eBiosciences). Cells were fixed and permeabilized using BD Cytofix/Cytoperm kit and then stained for TNF-α and IFN-γ. For Treg staining cells were labelled with a live dead marker and surface antibodies then intracellular staining of FoxP3 was performed using the FoxP3 mouse Treg cell staining kit (eBiosciences).

Flow cytometry was performed on a three-laser FACS aria or a four laser LSRII (BD Biosciences) in the Stanford Shared FACS Facility depending upon the experimental requirements. Analysis was performed on FlowJo software (Tree Star). Compensations were attained using compensation beads (Life Technologies) or cells stained with single stains of live dead stain. The following mouse Ab clones were used for analysis. Biolegend: CD45 (1:160, 103133, 30-F11), CD3 (1:80, 100237, 17A2), CD4 (1:400, 100406, GK1.5), CD8 (1:160, 100708, 53-6.7), CD11b (1:400, 101243, M1/70), CD11c (1:200, 117310, N418), F4/80 (1:60, 123110/123133, BM8), MHCII IA I-e (1:160, 107606, M5/114.15.2), GR-1 (1:160, 108408, RG6-8C5) and PD-L1 (1:160, 124310, 10 F.9G2). BD Biosciences: MHCI H2Kb (1:160, 553570, AF6-88.5), and Ki-67 (5ul per sample, 561126, B56). eBiosciences: FoxP3 (1:100, 61-5773, FJK165), IFN-γ (1:100, 11−7311, XMG1.2), TNF-α (1:100, 61−7321, MP6-XT22), and TCR-β (1:200, 45−5961, Η57−597). R&D Systems (MN): Axl (1:60, FAB8541P, 175128). Gating schemes of dissociated tumours was as follows: tumour cells (ZNIR^−^CD45^−^), CD8 T-cells (ZNIR^−^CD45^+^SSClowCD3^+^CD8^+^), CD4 T-cells (ZNIR^−^CD45^+^SSClowCD3^+^CD4^+^), macrophages (ZNIR^−^CD45^+^CD11b^+^F4/80^+^), immature myeloid cells (ZNIR^−^CD45^+^CD11b^+^GR1^+^), myeloid dendritic cells or antigen presenting myeloid cells (ZNIR^−^CD45^+^CD11b^+^CD11c^+^MHCII^+^) and CD4^+^ Tregs (AquaAmine^−^TCRβ^+^CD4^+^FoxP3^+^).

### Whole-exome sequencing

Mouse genomic DNA was extracted using QIAamp DNA Micro Kit (Qiagen) and 100 ng DNA was sonicated to an average size of 150–200 bp. The KAPA Library Preparation Kit (Kapa Biosystems) was used for library preparation, amplified with Illumina backbone oligonucleotides, and length was determined using a 2100 Bioanalyser (Agilent Technologies)^45^. SeqCap EZ Mouse Exome (Roche NimbleGen, Inc.) was used for sequence capture and four sample libraries were included in a single capture hybridization. DNA fragments were amplified with Illumina backbone oligonucleotides and cleaned with QIAquick PCR Purification Kit (Qiagen). Quality and yield of libraries was confirmed before sequencing on an Illumina HiSeq 2000 to a median depth of 42 × (Py117) and 33 × (Py8119). Paired-end reads were aligned to the mm9 reference genome using BWA-mem 0.7.5a. Variants were called using VarScan 2.3.6 with a minimum required depth of 10 reads, minimum of 3 variant supporting reads and minimum variant allele fraction of 10%. Variants called in both samples were excluded to remove germline single-nucleotide polymorphisms and potential recurrent mapping artefacts. Variants were post-filtered to include only coding changing (missense, stop-gain and frameshift or non-frameshift indel) variants with at least five variant supporting reads and at least 10% variant allele fraction.

### Protein isolation and western blotting

For protein analysis, cells were lysed with ice-cold RIPA buffer (50 mM Tris-HCl [pH 7.4], 150 mM NaCl, 1% NP-40, 0.1% SDS, 0.5% sodium deoxycholate) supplemented with protease and phosphatase inhibitor cocktails (Roche), then incubated on ice for 15 min, vortexed and centrifuged at 13,000 r.p.m. Protein lysates were quantified using BCA Protein Assay kit (Pierce), and 30–50 μg of protein samples were resolved by SDS–polyacrylamide gel electrophoresis according to standard methods, then transferred onto 0.2 μm supported nitrocellulose membranes (Bio-Rad Laboratories). The following primary antibodies were used to detect specific proteins: AXL (1:1,000; sc-1097, Santa Cruz Biotechnology), phospho-Axl (1:1,000; CST 5724, Cell Signaling technology), Gas6 (1:2,000; BAR-986, R&D), NF-κB p65 (1:1,000; sc-372, Santa Cruz Biotechnology), phospho-STAT1 (1:1,000; CST 7647, Cell Signaling Technologies), STAT1 (1:1,000; CST 9172, Cell Signaling technologies) HSP70 (1:2,000; #H5147, Sigma Aldrich), and β-Actin (1:5,000; #A5441, Sigma-Aldrich). Secondary antibodies used in this study were HRP-conjugated goat anti-mouse (1:10,000; Zymed Laboratories), HRP-conjugated goat anti-rabbit (1:5,000; #PI-1000, Vector Laboratories) and HRP-conjugated bovine anti-goat (1:5,000; sc-2350, Santa Cruz Biotechnology). Immunoblots were developed with SuperSignal West Dura Extended Duration Substrate (Thermo Fisher Scientific) and visualized with ChemiDoc XRS+ imaging system equipped with Image Lab Software (Bio-Rad Laboratories). Protein bands were quantified by densitometry using ImageJ software (National Institutes of Health). All gels from figures with protein markers visible are shown in Supplementary Fig. 12.

### Reverse phase protein array

For RPPA analysis, Py117 and Py8119 cells were trypsinized and resuspended in F12K media then plated 3 × 10^5^ in six-well plates. When suspended in 15 ml conical vial cells were radiated in a Cs irradiator to 10 Gy. After 24 h incubation cells were washed with PBS × 2 then 150 μl RIPA lysis buffer was added containing fresh protease and phosphatase inhibitor cocktail (Roche) and incubated on ice for 20 min. Cells were scraped collected and centrifuged at 13,000 r.p.m. for 10 min at 4 °C then the supernatant was isolated. Protein concentration was determined by BCA assay and diluted to a final concentration of 1 μg μl^−1^. Lysate was then mixed with 4 × SDS sample buffer containing 40% glycerol, 8% SDS, 0.24M Tris-HCl and 10% 2-mercaptoethanol. Samples were heated at 100 °C for 5 min and stored at −80 °C.

Samples were processed at the MD Anderson RPPA core facility according to their standard mouse protocol evaluating 281 mouse antibodies (https://www.mdanderson.org/education-and-research/resources-for-professionals/scientific-resources/core-facilities-and-services/functional-proteomics-rppa-core/index.html). Briefly, serial dilutions of lysates were arrayed on nitrocellulose-coated slides (Grace Biolabs) using an Aushon 2470 Arrayer (Aushon Biosystems) with a total of 5,808 spots were arranged per slide. Each slide was probed with a validated primary antibody plus a biotinylated secondary antibody. Antibodies had a Pearson correlation coefficient greater than 0.7 between RPPA and western blot. Signal was amplified using a Dako Cytomation-catalyzed system (Dako) and visualized by DAB colorimetric reaction then slides were scanned, analysed, and quantified using Microvigene software (Vigenetech Inc.) to generate spot intensity. Each dilution curve was fitted with a logistic model (http://bioinformatics.mdanderson.org/OOMPA) and fitted curve is plotted with observed and fitted signal intensities on the *y* axis and the Log_2_ concentration of proteins on the *x* axis. The protein concentrations of each set of slides were normalized by median polish, that was corrected across samples by the linear expression values using the median expression levels of all antibody experiments to calculate the loading correction for each sample.

A heatmap was generated from the Log_2_ values comparing Py117 and Py8119 with and without radiation. EMT associated proteins differentially expressed between the two cell lines were then plotted in a heatmap for comparison of the pertinent differences identified.

### Development of CRISPR Py8119 clones

To construct Axl CRISPR clones, Py8119 cells were transfected with the mCMV promoter driving Edit-R Cas9 expression plasmid with a puromycin resistance marker (GE Life Sciences) using the DharmaFECT Duo Transfection Reagent (GE Life Sciences). After 72 h, cells were treated with 6 μg ml^−1^ puromycin for 5 days. Cells growing under selection were then transfected with Axl targeting crRNA (GCGCCAACCACCAGGCCAGCGUUUUAGAGCUAUG CUGUUUUG) and tracrRNA (GE Life Sciences) using Dharmafect Duo transfection reagent. After 5–7 days cells were analysed by flow cytometry to verify population of cells with absent Axl surface expression. Cells with no Axl expression were sorted, single cell dilutions were plated in 96-well plate, clones were grown for 10–14 days and clones were screened for Axl expression by flow cytometry.

### Luminex multiplex cytokine assay

Py117 cells, Py8119 vector control, Axl Cr #1 and #2 were plated in 6 cm dishes with 3.33 × 10^5^ cells. At 24 h media was changed and then collected after 48 h, passed through a 0.2 μm filter, and concentrated with an Amicon Ultra Centrifugal filters with 3 kDa cutoff (Millipore). Conditioned media was stored at −80 °C until processing. Triplicate samples for each cell line were plated and collected independently on different days.

Mouse 38-plex kits were purchased form Affymetrix and used according to manufacturer's recommendations. The Stanford Human Immune Monitoring Core processed samples as follows. Antibody linked beads were added to a 96-well plate washed with Biotech Elx405 washer and 60 μl of concentrated conditioned media samples were added and incubated for 1h at room temperature followed by overnight incubation at 4 °C. Plates were washed and biotinylated detection antibody was added for 75 min at room temperature with shaking. After a wash, streptavidin PE was added for 30 min. The plate was washed and resuspended in reading buffer was added and each sample was measured in duplicate. Plates were read using a Luminex 200 instrument (Luminex Corp.) with a lower bound of 50 beads per sample cytokine. Custom assay Control beads (Radix Biosolutions) were added to all wells. All sample values within a standard curve with lower limit of 0.43–37.22 pg ml^−1^ and upper limit of 0.7–150 ng ml^−1^ and that had higher values than culture media with 5% serum were included for analysis. Mean fluorescence intensity was used for comparison due to different buffers used for standards.

### Real-time PCR

RNA was isolated using Trizol (Invitrogen) and subsequently treated with DNase I (Fermentas). First-strand cDNA synthesis was performed with SuperScript II Reverse Transcriptase and random primers (Invitrogen) according to the manufacturer's instructions. Quantitative real-time PCR was carried out using Power SYBR Green Master Mix (Life Technologies), detection and data analysis were executed with the 7900HT Fast Real-Time PCR System (Applied Biosystems) by computing the results relative to a standard curve made with cDNA pooled from all samples, normalized to 18S. Primer sequences used to amplify specific target genes were obtained from the Universal ProbeLibrary (https://lifescience.roche.com/en_us/brands/universal-probe-library.html). Mouse MHCI mRNA was amplified using forward primer 5′-GGAAAAGGAGGGGACTATGCT-3′ and reverse primer 5′-GAGGGTCATGAACCATCACTTT-3′. NF-kB-p65 using forward primer 5′-CTCAACTTCTGTCCCCAAGC-3′ and reverse primer 5′- TGGGGGAAAACTCATCAAAG-3′. IL-6 using forward primer 5′-TCTAATTCATATCTTCAACCAAGAGG-3′ and reverse primer 5′- TGGTCCTTAGCCACTCCTTC-3′. Lastly CIITA measured using forward primer 5′- GATGGATGTCCAGTTCAACAAG-3′ and reverse primer 5′-GGATAGTGGGTGTCCACATTG-3′.

### TCGA data analysis

The TCGA breast invasive carcinoma data set was accessed through the UCSC Cancer Browser (https://Genome-cancer.ucsc.edu/proj/site/hgHeatmap/)^30^. RNAseq gene expression values from 1,215 patients in the data set performed on the IlluminaHiSeq platform were obtained for the genes identified in Fig. 4o. Gene signatures for PyMT Py8119 cell Axl associated genes (PyMT signature) and an EMT signature adapted from Byers *et al*. and are listed in Fig. 4o (ref. 31). Correlation analysis was performed using Pearson and Spearman tests using Prism 6 software (Graphpad Software Inc.). The same analysis was performed with the Pan-Cancer dataset containing human patient sequence expression data for 9,755 patients with all types of cancers in the TCGA and the Cancer Cell Line Encyclopedia published in the UCSC Cancer Browser database.

### Statistics

Clonogenic survival, flow cytometry, cell, molecule, and tumour analysis, and Luminex cytokine assays were analysed in an analysis of variance model and post hoc comparison was performed using a Tukey or Dunnett adjustment as appropriate. Growth curves were analysed in a repeated measures model allowing for errors to be correlated within mice. Post hoc pairwise comparisons were performed using a Tukey adjustment. Mouse survival was summarized using Kaplan-Meier curves and tested using a log-rank test. All analyses were performed using SAS9.4 (SAS Institute Inc, NC) or GraphPad6 (Software Inc, CA).

### Data availability

All relevant data are available from the authors.

## Additional information

**How to cite this article:** Aguilera, T. A. *et al*. Reprogramming the immunological microenvironment through radiation and targeting Axl. *Nat. Commun.* **7,** 13898 doi: 10.1038/ncomms13898 (2016).

**Publisher's note:** Springer Nature remains neutral with regard to jurisdictional claims in published maps and institutional affiliations.

## Supplementary Material

Supplementary Information Supplementary Figures

Supplementary Data 1 Unique mutations identified through whole exome next generation sequencing of Py117 and Py8119 tumor cells. The table shows all unique mutations resulting in stop-gain, frameshift or nonsynonymous variants. Each mutation has the accompanying information: chr= chromosome, Pos [mm9]= position of the mutation on the reference mouse genome, Ref= reference nucleotide, Var= variant, Gene= gene affected, Type= the result of the mutation (stopgain, frameshift, or nonsynonymous), Amino Acid Change= impact on amino acid sequence, Total Depth= depth of the sequencing, Var Depth= depth of the identified variant, VAF%= percent of variant frequency.

## References

[b1] Postow M. A., Callahan M. K. & Wolchok J. D. Immune checkpoint blockade in cancer therapy. J. Clin. Oncol. 33, 1974–1982 (2015). 25605845 10.1200/JCO.2014.59.4358 PMC4980573

[b2] Hodi F. S. . Improved survival with ipilimumab in patients with metastatic melanoma. N. Engl. J. Med. 363, 711–723 (2010). 20525992 10.1056/NEJMoa1003466 PMC3549297

[b3] Brahmer J. R. . Safety and activity of anti-PD-L1 antibody in patients with advanced cancer. N. Engl. J. Med. 366, 2455–2465 (2012). 22658128 10.1056/NEJMoa1200694 PMC3563263

[b4] Topalian S. L. . Safety, activity, and immune correlates of anti-PD-1 antibody in cancer. N. Engl. J. Med. 366, 2443–2454 (2012). 22658127 10.1056/NEJMoa1200690 PMC3544539

[b5] Robert C. . Ipilimumab plus dacarbazine for previously untreated metastatic melanoma. N. Engl. J. Med. 364, 2517–2526 (2011). 21639810 10.1056/NEJMoa1104621

[b6] Postow M. A. . Immunologic correlates of the abscopal effect in a patient with melanoma. N. Engl. J. Med. 366, 925–931 (2012). 22397654 10.1056/NEJMoa1112824 PMC3345206

[b7] Hiniker S. M. . A systemic complete response of metastatic melanoma to local radiation and immunotherapy. Transl. Oncol. 5, 404–407 (2012). 23323154 10.1593/tlo.12280 PMC3542835

[b8] Slovin S. F. . Ipilimumab alone or in combination with radiotherapy in metastatic castration-resistant prostate cancer: results from an open-label, multicenter phase I/II study. Ann. Oncol. 24, 1813–1821 (2013). 23535954 10.1093/annonc/mdt107 PMC3707423

[b9] Twyman-Saint Victor C. . Radiation and dual checkpoint blockade activate non-redundant immune mechanisms in cancer. Nature 520, 373–377 (2015). 25754329 10.1038/nature14292 PMC4401634

[b10] Golden E. B., Demaria S., Schiff P. B., Chachoua A. & Formenti S. C. An abscopal response to radiation and ipilimumab in a patient with metastatic non-small cell lung cancer. Cancer Immunol. Res. 1, 365–372 (2013). 24563870 10.1158/2326-6066.CIR-13-0115 PMC3930458

[b11] Grimaldi A. M. . Abscopal effects of radiotherapy on advanced melanoma patients who progressed after ipilimumab immunotherapy. Oncoimmunology 3, e28780 (2014). 25083318 10.4161/onci.28780 PMC4106166

[b12] Deng L. . Irradiation and anti-PD-L1 treatment synergistically promote antitumor immunity in mice. J. Clin. Invest. 124, 687–695 (2014). 24382348 10.1172/JCI67313 PMC3904601

[b13] Sharabi A. B. . Stereotactic radiation therapy augments antigen-specific PD-1-mediated antitumor immune responses via cross-presentation of tumor antigen. Cancer Immunol. Res. 3, 345–355 (2015). 25527358 10.1158/2326-6066.CIR-14-0196 PMC4390444

[b14] Reits E. A. . Radiation modulates the peptide repertoire, enhances MHC class I expression, and induces successful antitumor immunotherapy. J. Exp. Med. 203, 1259–1271 (2006). 16636135 10.1084/jem.20052494 PMC3212727

[b15] Dovedi S. J. . Acquired resistance to fractionated radiotherapy can be overcome by concurrent PD-L1 blockade. Cancer Res. 74, 5458–5468 (2014). 25274032 10.1158/0008-5472.CAN-14-1258

[b16] Spranger S., Bao R. & Gajewski T. F. Melanoma-intrinsic beta-catenin signalling prevents anti-tumour immunity. Nature 523, 231–235 (2015). 25970248 10.1038/nature14404

[b17] Kim K. . Eradication of metastatic mouse cancers resistant to immune checkpoint blockade by suppression of myeloid-derived cells. Proc. Natl Acad. Sci. USA 111, 11774–11779 (2014). 25071169 10.1073/pnas.1410626111 PMC4136565

[b18] Finisguerra V. . MET is required for the recruitment of anti-tumoural neutrophils. Nature 522, 349–353 (2015). 25985180 10.1038/nature14407 PMC4594765

[b19] Zhang Z. . Activation of the AXL kinase causes resistance to EGFR-targeted therapy in lung cancer. Nat. Genet. 44, 852–860 (2012). 22751098 10.1038/ng.2330 PMC3408577

[b20] Rankin E. B. . Direct regulation of GAS6/AXL signaling by HIF promotes renal metastasis through SRC and MET. Proc. Natl Acad. Sci. USA 111, 13373–13378 (2014). 25187556 10.1073/pnas.1404848111 PMC4169907

[b21] Graham D. K., DeRyckere D., Davies K. D. & Earp H. S. The TAM family: phosphatidylserine sensing receptor tyrosine kinases gone awry in cancer. Nat. Rev. Cancer 14, 769–785 (2014). 25568918 10.1038/nrc3847

[b22] Hugo W. . Genomic and transcriptomic features of response to anti-PD-1 therapy in metastatic melanoma. Cell 165, 35–44 (2016). 26997480 10.1016/j.cell.2016.02.065 PMC4808437

[b23] Youn J. I., Nagaraj S., Collazo M. & Gabrilovich D. I. Subsets of myeloid-derived suppressor cells in tumor-bearing mice. J. Immunol. 181, 5791–5802 (2008). 18832739 10.4049/jimmunol.181.8.5791 PMC2575748

[b24] Peggs K. S., Quezada S. A., Chambers C. A., Korman A. J. & Allison J. P. Blockade of CTLA-4 on both effector and regulatory T cell compartments contributes to the antitumor activity of anti-CTLA-4 antibodies. J. Exp. Med. 206, 1717–1725 (2009). 19581407 10.1084/jem.20082492 PMC2722174

[b25] Dewan M. Z. . Fractionated but not single-dose radiotherapy induces an immune-mediated abscopal effect when combined with anti-CTLA-4 antibody. Clin. Cancer Res. 15, 5379–5388 (2009). 19706802 10.1158/1078-0432.CCR-09-0265 PMC2746048

[b26] Rothlin C. V., Ghosh S., Zuniga E. I., Oldstone M. B. & Lemke G. TAM receptors are pleiotropic inhibitors of the innate immune response. Cell 131, 1124–1136 (2007). 18083102 10.1016/j.cell.2007.10.034

[b27] Sen P. . Apoptotic cells induce Mer tyrosine kinase-dependent blockade of NF-kappaB activation in dendritic cells. Blood 109, 653–660 (2007). 17008547 10.1182/blood-2006-04-017368 PMC1785106

[b28] Camenisch T. D., Koller B. H., Earp H. S. & Matsushima G. K. A novel receptor tyrosine kinase, Mer, inhibits TNF-alpha production and lipopolysaccharide-induced endotoxic shock. J. Immunol. 162, 3498–3503 (1999). 10092806

[b29] Pollack B. P., Sapkota B. & Cartee T. V. Epidermal growth factor receptor inhibition augments the expression of MHC class I and II genes. Clin. Cancer Res. 17, 4400–4413 (2011). 21586626 10.1158/1078-0432.CCR-10-3283

[b30] Cline M. S. . Exploring TCGA Pan-Cancer data at the UCSC Cancer Genomics Browser. Sci. Rep. 3, 2652 (2013). 24084870 10.1038/srep02652 PMC3788369

[b31] Byers L. A. . An epithelial-mesenchymal transition gene signature predicts resistance to EGFR and PI3K inhibitors and identifies Axl as a therapeutic target for overcoming EGFR inhibitor resistance. Clin. Cancer Res. 19, 279–290 (2013). 23091115 10.1158/1078-0432.CCR-12-1558 PMC3567921

[b32] Merad M., Sathe P., Helft J., Miller J. & Mortha A. The dendritic cell lineage: ontogeny and function of dendritic cells and their subsets in the steady state and the inflamed setting. Annu. Rev. Immunol. 31, 563–604 (2013). 23516985 10.1146/annurev-immunol-020711-074950 PMC3853342

[b33] Tiemessen M. M. . CD4^+^CD25^+^Foxp3^+^ regulatory T cells induce alternative activation of human monocytes/macrophages. Proc. Natl Acad. Sci. USA 104, 19446–19451 (2007). 18042719 10.1073/pnas.0706832104 PMC2148309

[b34] Liu G. . Phenotypic and functional switch of macrophages induced by regulatory CD4^+^CD25^+^ T cells in mice. Immunol. Cell Biol. 89, 130–142 (2011). 20514074 10.1038/icb.2010.70

[b35] Kariolis M. S. . An engineered Axl ‘decoy receptor' effectively silences the Gas6-Axl signaling axis. Nat. Chem. Biol. 10, 977–983 (2014). 25242553 10.1038/nchembio.1636 PMC4372605

[b36] Ye X. . An anti-Axl monoclonal antibody attenuates xenograft tumor growth and enhances the effect of multiple anticancer therapies. Oncogene 29, 5254–5264 (2010). 20603615 10.1038/onc.2010.268

[b37] Sheridan C. First Axl inhibitor enters clinical trials. Nat. Biotechnol. 31, 775–776 (2013). 24022140 10.1038/nbt0913-775a

[b38] Liu L. . Novel mechanism of lapatinib resistance in HER2-positive breast tumor cells: activation of AXL. Cancer Res. 69, 6871–6878 (2009). 19671800 10.1158/0008-5472.CAN-08-4490

[b39] Zagorska A., Traves P. G., Lew E. D., Dransfield I. & Lemke G. Diversification of TAM receptor tyrosine kinase function. Nat. Immunol. 15, 920–928 (2014). 25194421 10.1038/ni.2986 PMC4169336

[b40] Paolino M. . The E3 ligase Cbl-b and TAM receptors regulate cancer metastasis via natural killer cells. Nature 507, 508–512 (2014). 24553136 10.1038/nature12998 PMC6258903

[b41] Alciato F., Sainaghi P. P., Sola D., Castello L. & Avanzi G. C. TNF-alpha, IL-6, and IL-1 expression is inhibited by GAS6 in monocytes/macrophages. J. Leukoc. Biol. 87, 869–875 (2010). 20103767 10.1189/jlb.0909610

[b42] Paccez J. D. . The receptor tyrosine kinase Axl is an essential regulator of prostate cancer proliferation and tumor growth and represents a new therapeutic target. Oncogene 32, 689–698 (2013). 22410775 10.1038/onc.2012.89 PMC4078100

[b43] Davie S. A. . Effects of FVB/NJ and C57Bl/6J strain backgrounds on mammary tumor phenotype in inducible nitric oxide synthase deficient mice. Transgenic Res. 16, 193–201 (2007). 17206489 10.1007/s11248-006-9056-9 PMC1829418

[b44] Bao L., Cardiff R. D., Steinbach P., Messer K. S. & Ellies L. G. Multipotent luminal mammary cancer stem cells model tumor heterogeneity. Breast Cancer Res. 17, 137 (2015). 26467658 10.1186/s13058-015-0615-y PMC4606989

[b45] Newman A. M. . An ultrasensitive method for quantitating circulating tumor DNA with broad patient coverage. Nat. Med. 20, 548–554 (2014). 24705333 10.1038/nm.3519 PMC4016134

