# Supplementary material for: Reprogramming the immunological microenvironment through radiation and targeting Axl
Source: Nat Commun. 2016 Dec 23;7:13898. doi: 10.1038/ncomms13898 (PMC5196438; doi:10.1038/ncomms13898)
Supplement: Supplementary Information — Supplementary Figures [file ncomms13898-s1.pdf]

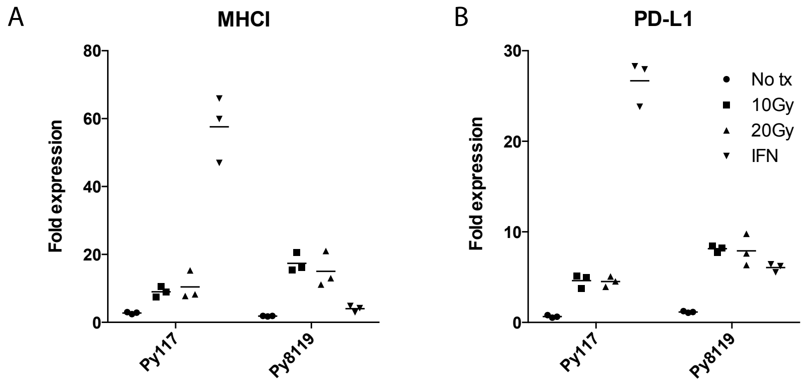

Supplementary Figure 1. Radiation and IFN-γ enhance MHCI expression and PD-L1 on PyMT tumor cells but Py117 cells are more responsive to IFN-γ. (**A**) Flow cytometry of MHCI expression in Py117 and Py8119 cells after 10 Gy, 20 Gy, or IFN-γ. Both tumors respond with greater MHCI expression after RT but there is little MHCI response to IFN-γ in the Py8119 cells. (**B**) Similarly PD-L1 is increased in both cell lines after radiation but to a much less extent than after IFN−γ stimulation (bar represents mean).

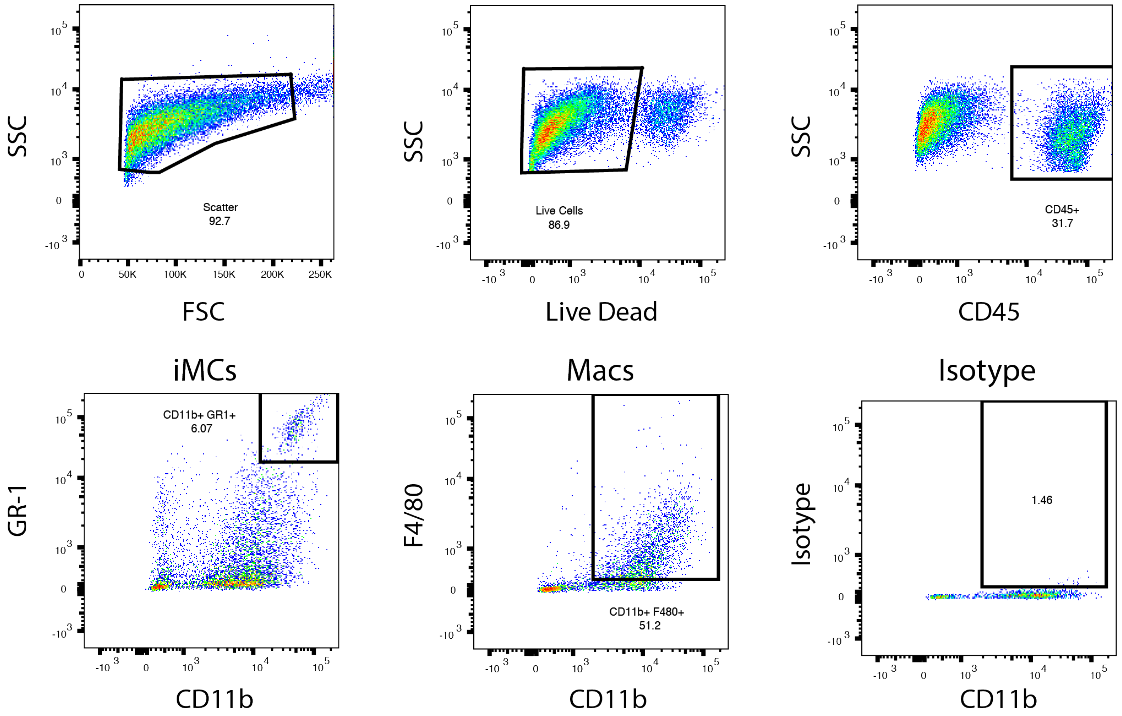

Supplementary Figure 2. Gating strategy of infiltrating leukocytes by flow cytometry. The scatter, live cell, and CD45 gates are shown from PyMT infiltrating leukocytes. Immature myeloid cells (CD45^{+}CDllb^{+}Gr1^{Hi}) are shown in the bottom left panel showing the small population with very high GR1 expression. The lower middle panel shows the macrophage panel (CD45^{+}CDllb^{+}F4/80^{+}) and the lower right shows isotype control with CD11b^{+} cells.

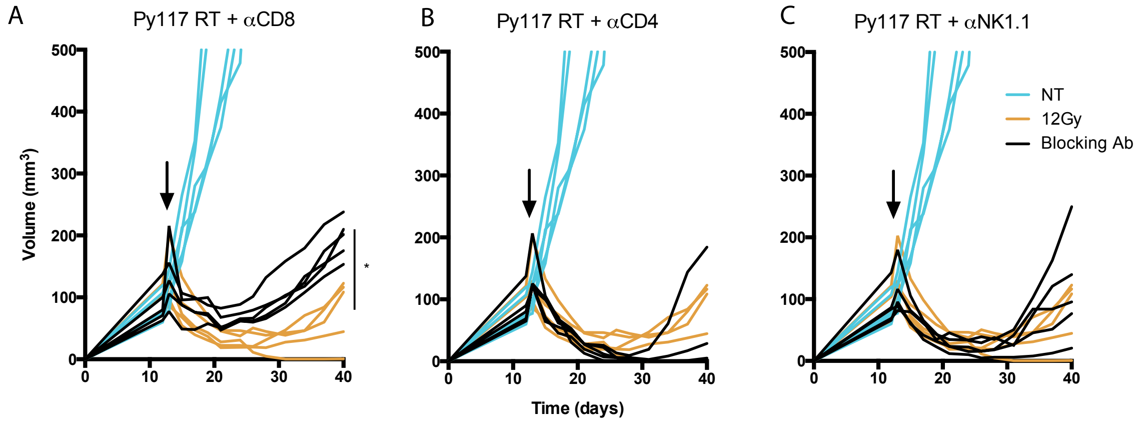

Supplementary Figure 3. CD8$^+$ T cells are important to radiation response. (A) Radiation (RT) response after 12 Gy was diminished in wild type C57BL/6 mice treated with CD8 blocking antibody (black curves) compared to 12 Gy of RT with isotype Ab (orange)(*p=0004). **(B)** Blocking CD4 T cells lead to a greater response than RT alone. **(C)** There was no difference in radiation response when blocking NK cells with the NK1.1 Ab suggesting NK cells do not play a rule in the immune response. (5 mice each, isotype control mice that did not receive radiation are shown in blue; 12 Gy RT was given 13 days after implantation.)

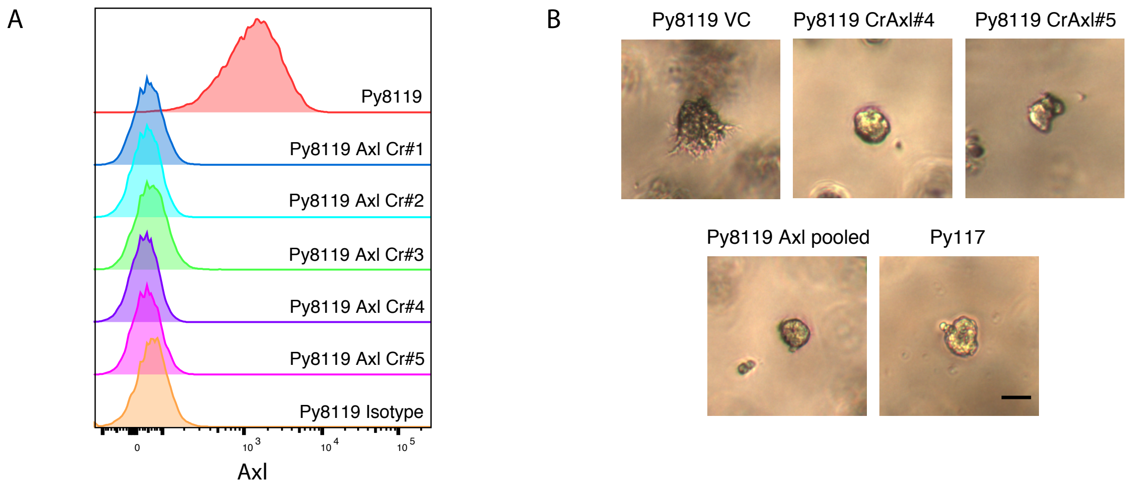

Supplementary Figure 4. Surface expression of Axl was successfully knocked out in CRISPR clones of Py8119 tumor cells which impacts invasiveness in 3D culture. (A) Flow cytometry of Axl surface expression on wild type Py8119 cells and 5 different Axl CRISPR clones stimulated with IFN-γ to confirm knockout. **(B)** Compared to Py8119 vector controlled tumor cells Axl Cr#4, Cr#5, pooled Axl Cr, and Py117 have less invasion into matrigel 4 days after plating (representative phase contrast images, scale bars = 50 μm).

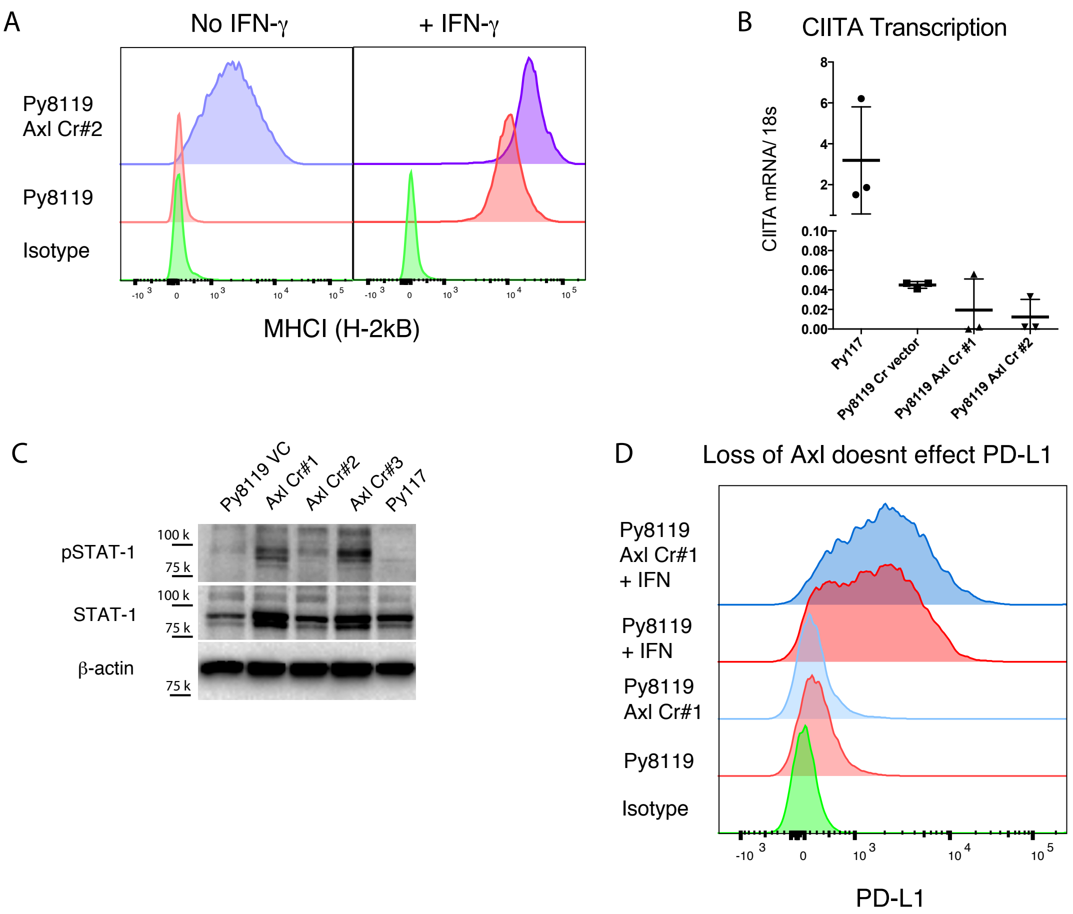

Supplementary Figure 5. Loss of Axl leads to increase in MHCI but no change in PD-L1. **(A)** In the absence of IFN- γ stimulation MHCI expression is increased in the CRISPR Axl Cr#2 cells compared to parental Py8119 cells. After overnight stimulation with 25 ng/mL of IFN- γ MHCI is further is induced compared to parental IFN-γ stimulated cells. **(B)** qRT-PCR of CIITA MHCI co-activator reveals no change in transcription between Py8119 vector control cells compared to Axl Cr#1 and Cr#2 (Bars= mean and ±s.d.). **(C)** Western blot of Py8119 VC, 3 different Py8119 Axl Cr clones, and Py117 cells reveals greater pSTAT-1 and STAT-1 levels in knockout cells. **(D)** Expression of PD-L1 in Py8119 parental cells in culture is not influenced by Py8119 Axl Cr#1 knockout cells and remains inducible by IFN- γ.

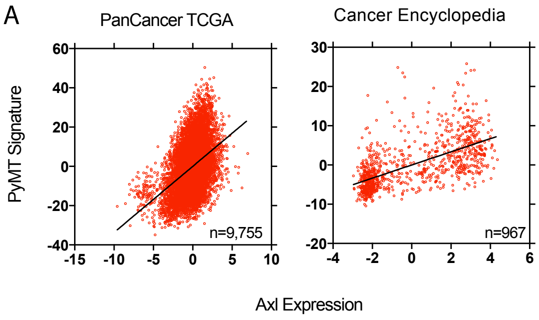

B

Disease	Pearson r	Spearman r
PanCancer	0.42	0.4
Head and Neck	0.46	0.44
Lung (SCC/AC)	0.57	0.57
Melanoma	0.57	0.57
Cervical	0.56	0.51
Prostate	0.64	0.63
Liver	0.71	0.71
Cancer Encyclopedia	0.61	0.66

Supplementary Figure 6. The PyMT signature significantly correlates with Axl expression in the Pan-Cancer TCGA and Cancer Cell Line Encyclopedia. (A) RNAseq gene expression of the PyMT signature correlation with Axl was significant in the Pan-Cancer RNAseq data set with 9,755 patient samples and the Cancer Cell Line Encyclopedia of all types of cancer cell line gene expression. **(B)** Pearson and Spearman r-values were calculated for these data sets are listed as well as a selection of certain tumor types from the Pan-Cancer data set ($p<0.0001$ for all correlations).

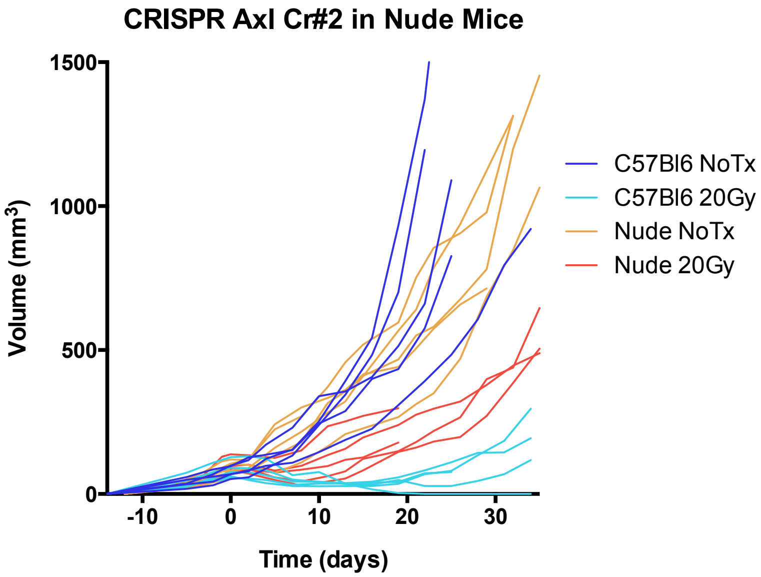

Supplementary Figure 7. Py8119 Axl Cr#2 tumors are less responsive to radiation when implanted into immunodeficient nude mice. Py8119 Axl Cr#2 tumors were implanted into wild type C57Bl6 mice and athymic nude mice, and then treated with 20 Gy of radiation 12-14 days after implantation. Growth curves reveal that the radiation response is suppressed in nude mice confirming the importance of a T cell response in the radiosensitivity of the Py8119 Axl Cr#2 tumors (5 mice per treatment group).

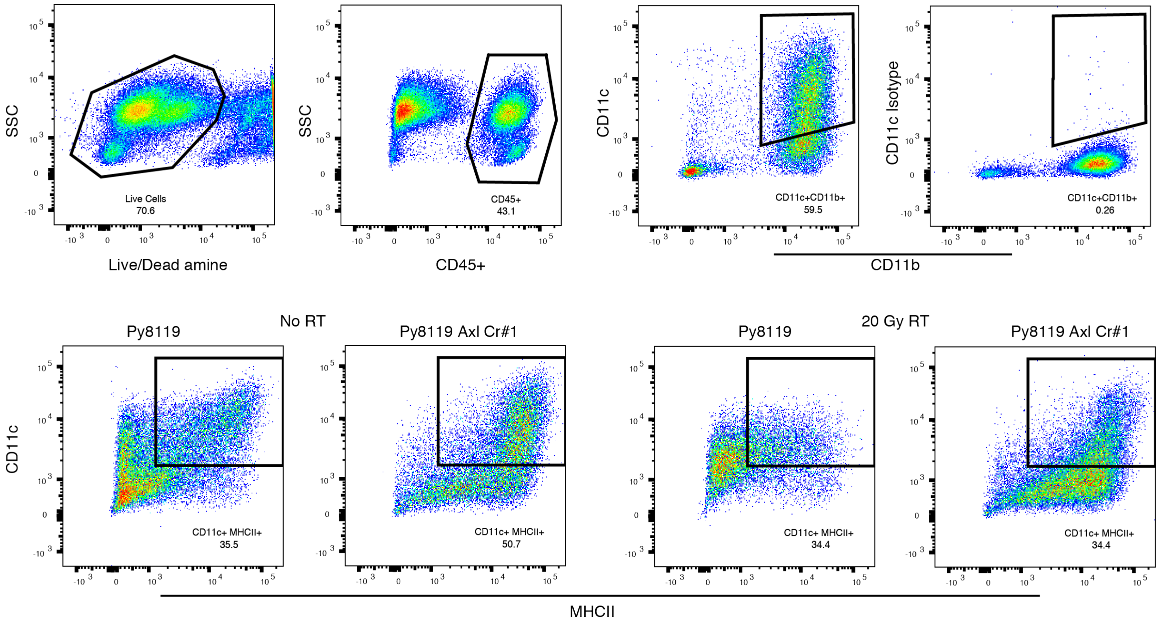

Supplementary Figure 8. Gating scheme for myeloid dendritic cells, $CD11b^{+}CD11c^{+}MHCII^{+}$.

Cells were isolated, labeled, and then gated by flow cytometry on live cells, $CD45^{+}$ cells, and $CD11b^{+}CD11c^{+}$, and $MHCII^{+}$. Isotype controls are included for comparison. Representative gates on $CD11c^{+}MHCII^{+}$ cells are shown.

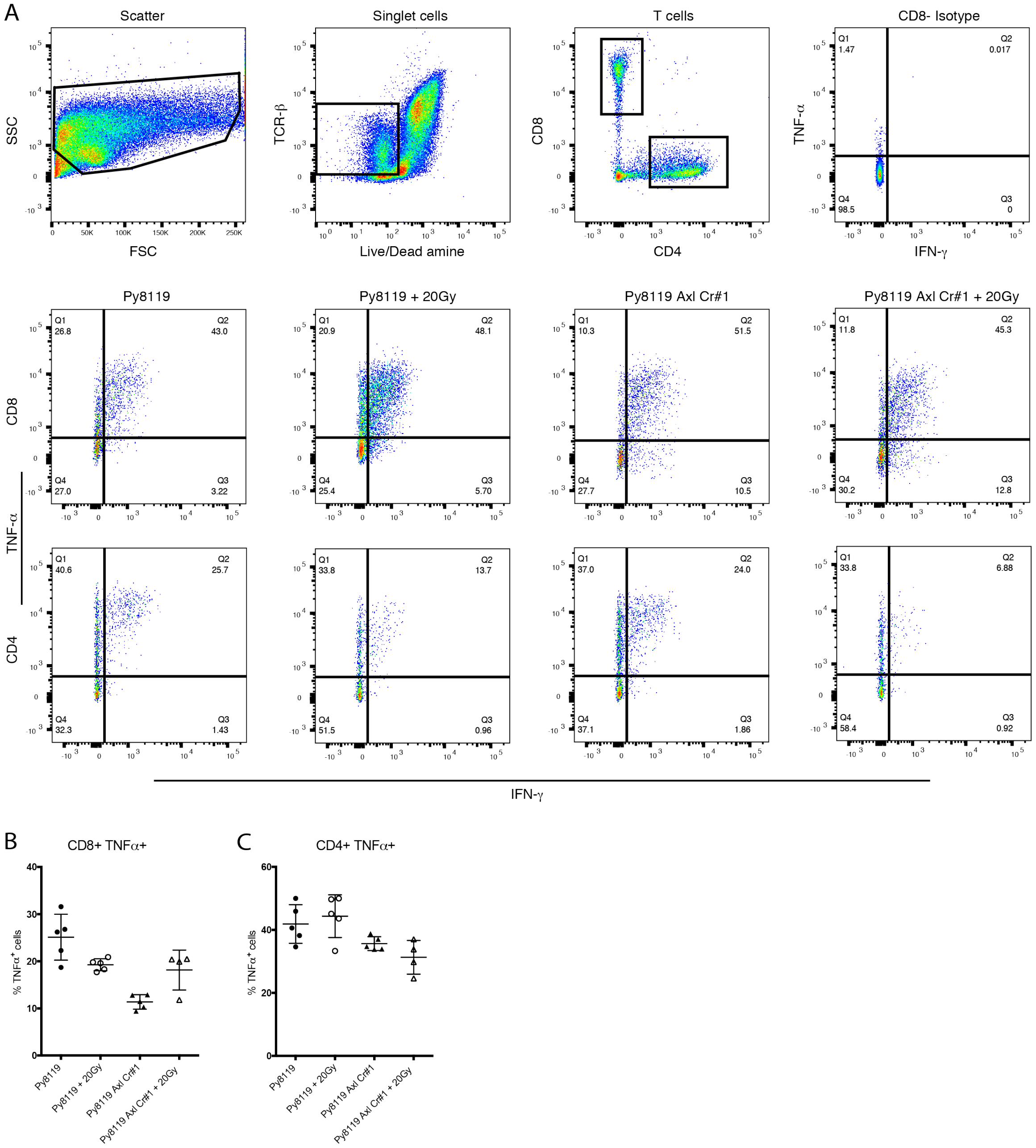

Supplementary Figure 9. Gating scheme for functional T cell assay to interrogate cytokine expression by flow cytometry. (A) Scatter gate, TCRβ/live dead cell gate, and T cell gates are shown in upper panels. TNFα and IFN-γ labeling for flow cytometry reveal a significant proportion of cytokine producing cells. Quantitative data for the proportion of $CD8^+$ T cells (B) and $CD4^+$ T cells (C) expressing high levels TNF-α and absent IFN-γ.

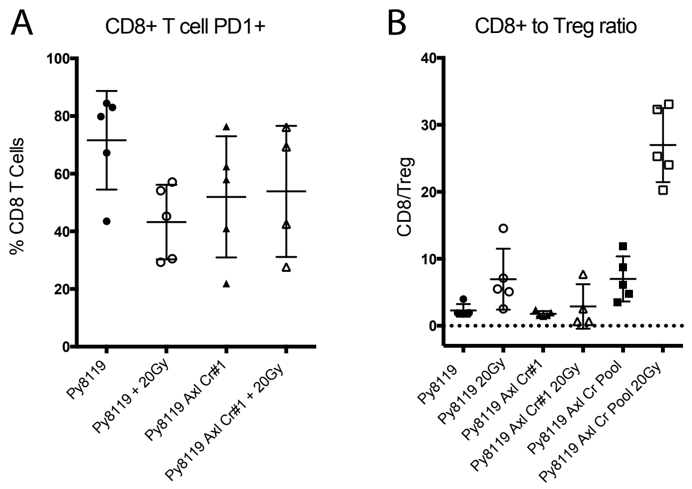

Supplementary Figure 10. T cells are functionally active in Axl knockout tumors with an effector phenotype. (A) PD-1 expression on $CD8^+$ T cells reveals little change in Axl Cr#1 tumors with and without radiation compared to Py8119. The proportion of $PD\text{-}1^+$ cells is higher in the untreated Py8119 tumors. **(B)** The $CD8^+$ T cells to T reg ratio is elevated in after radiation and much greater in Axl Cr pool tumors suggesting greater antitumor activity at the 10 day time point in CRISPR pool tumors compared to wild type tumors and Axl Cr#1 tumors.

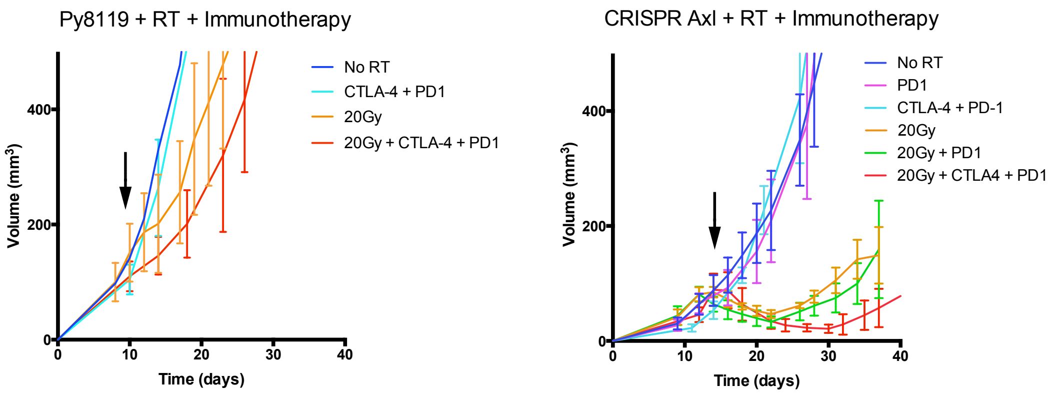

Supplementary Figure 11. Radiation and immunotherapy is effective in Axl knockout tumors but not wild type Py8119 tumors. The combination of PD-1 + CTLA-4 + 20 Gy RT in Axl Cr#1 tumors is sufficient for improved antitumor response after radiation (right panel from Fig. 5) compared to little to no response in Py8119 parental tumors. Mice were treated with 20 Gy on day 10 for Py8119 tumors and 20 Gy on day 14 for Py8119 Axl Cr#1 when the tumors were similar size. (5 mice per treatment group)

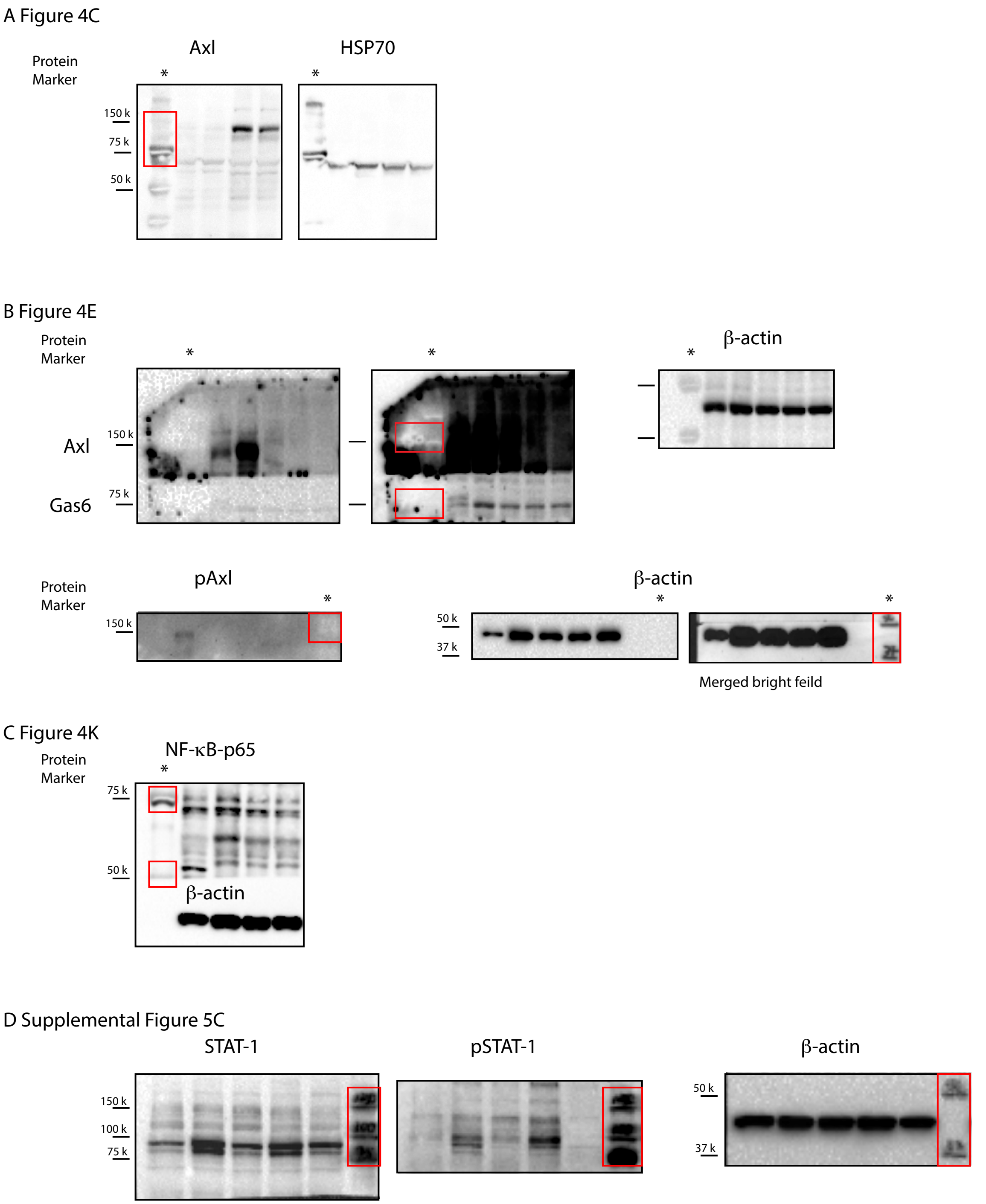

Supplementary Figure 12. Western Blot analysis with protein markers. All western blots in the figures and supplementary figures are displayed here with a wider view and depicting evidence of protein markers (labeled with a red box). **(A)** Shows blots from Figure 4C, **(B)** from Figure 4E, **(C)** from Figure 4K, and **(D)** Supplementary Figure 5C.
